# Distant Metastases of Breast Cancer Resemble Primary Tumors in Cancer Cell Composition but Differ in Immune Cell Phenotypes

**DOI:** 10.1158/0008-5472.CAN-24-1211

**Published:** 2024-10-22

**Authors:** Laura Kuett, Alina Bollhagen, Sandra Tietscher, Bettina Sobottka, Nils Eling, Zsuzsanna Varga, Holger Moch, Natalie de Souza, Bernd Bodenmiller

**Affiliations:** 1 Department of Quantitative Biomedicine, University of Zurich, Zurich, Switzerland.; 2 Institute of Molecular Health Sciences, ETH Zurich, Zurich, Switzerland.; 3 Life Science Zurich Graduate School, ETH Zurich and University of Zurich, Zurich, Switzerland.; 4 Department of Pathology and Molecular Pathology, University and University Hospital Zurich, Zurich, Switzerland.; 5 Institute of Molecular Systems Biology, ETH Zurich, Zurich, Switzerland.

## Abstract

Breast cancer is the most commonly diagnosed cancer in women, with distant metastasis being the main cause of breast cancer–related deaths. Elucidating the changes in the tumor and immune ecosystems that are associated with metastatic disease is essential to improve understanding and ultimately treatment of metastasis. Here, we developed an in-depth, spatially resolved single-cell atlas of the phenotypic diversity of tumor and immune cells in primary human breast tumors and matched distant metastases, using imaging mass cytometry to analyze a total of 75 unique antibody targets. Although the same tumor cell phenotypes were typically present in primary tumors and metastatic sites, suggesting a strong founder effect of the primary tumor, their proportions varied between matched samples. Notably, the metastatic site did not influence tumor phenotype composition, except for the brain. Metastatic sites exhibited a lower number of immune cells overall but had a higher proportion of myeloid cells as well as exhausted and cytotoxic T cells. Myeloid cells showed distinct tissue-specific compositional signatures and increased presence of potentially matrix remodeling phenotypes in metastatic sites. This analysis of tumor and immune cell phenotypic composition of metastatic breast cancer highlights the heterogeneity of the disease within patients and across distant metastatic sites, indicating myeloid cells as the predominant immune modulators that could potentially be targeted at these sites.

**Significance:**

Multiplex imaging analysis of matched primary and metastatic breast tumors provides a phenotypic and spatial map of tumor microenvironments, revealing similar compositions of cancer cells and divergent immunologic features between matched samples.

## Graphical Abstract

**Figure:**
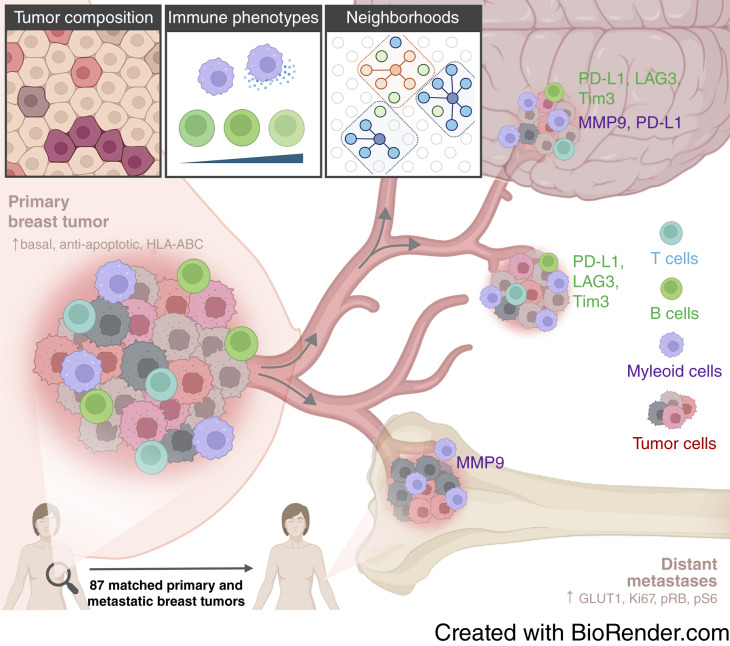

## Introduction

Breast cancer is the most frequently diagnosed cancer in women (1). Clinically, breast cancer is classified into four major subtypes [luminal A and B, HER2, and triple-negative (TN)] based on the expression of hormone receptors, namely estrogen receptor (ER) and progesterone receptor, HER2, and the proliferation marker Ki67. Overall survival varies between clinical subtypes, but all have in common that the development of distant metastasis is the primary cause of death, with a 5-year survival rate of 29% for metastatic disease (2). Treatment decisions for metastatic breast cancer are primarily based on histologic and molecular assessment of the primary tumor (PT; ref 3). Only recently, treatment guidelines recommend that distant metastasis should be sampled to determine differences in molecular diagnostic markers between primary and metastatic lesions (4).

For metastatic breast cancer, a combination of targeted therapies is used alongside chemotherapy, with the most effective first-line treatment depending on the subtype and genetic background (4, 5). However, response durations are often limited, and there is still a lack of understanding of targetable immune features at metastatic sites. Thorough examination of matched breast cancer PT and metastases would contribute toward better understanding of metastatic disease and may improve disease management.

The few studies so far comparing matched PT and metastases have only identified small differences. Genomic analysis of tumor cells revealed a slight elevation in the frequency of disease-relevant somatic variations (e.g., in *TP53*, *PTEN*, and *RB1*) and of DNA amplifications/deletions in metastatic tumors, and minimal changes in the mutation pattern, relative to PT (6–10). Bulk RNA sequencing analysis revealed that in about 30% of cases, the expression subtype changed from primary to the metastatic tumor (10–13), consistent with IHC studies of clinical markers (14–17). Multiomic analysis showed fewer fibroblasts and endothelial cells in metastatic luminal disease and less B and T cells in metastatic TN breast cancer compared with PT (10), whereas anti-inflammatory macrophages were significantly increased across all distant sites (18). Furthermore, we previously showed that CD8^+^ T-cell infiltration patterns, including expression of PD-1 and LAG3, tend to be shared between primary and metastatic tumors (19). What is currently missing, however, is a single-cell analysis that resolves tumor and immune phenotypes at matched primary and distant metastatic sites, which could reveal metastasis specific drug targets.

We and others have analyzed primary breast tumors using single-cell transcriptomics (20), single-cell protein analysis (21), and highly multiplexed tissue imaging (22–26), including a comparison of PT and lymph node metastasis (27). These studies have uncovered phenotypically diverse tumor cell populations that can be used to identify patient subgroups with varying survival outcomes (23). Extending the concept of tumor phenotypes to the investigation of metastatic disease could unravel targetable features and advance the biological understanding of metastatic disease.

Here, we used imaging mass cytometry (IMC; ref. 28) on 75 unique antibody targets to study tumor and immune cell composition of matched primary and distant metastatic tumors in formalin-fixed paraffin-embedded samples from 87 individuals with metastatic breast cancer. The cohort included all the major breast cancer metastatic sites except lung and covered all disease subtypes. Tumor cell phenotypes were largely shared between PT and metastases, but the extent of compositional similarity between matched samples varied widely across patients, independent of metastatic tissue site. We observed a higher proportion of antigen-experienced and cytotoxic T cells in metastases compared with their matched PT. Furthermore, macrophages were enriched in metastases, with a higher proportion of potentially matrix remodeling phenotypes, and showed metastasis location-specific phenotypes. Our analysis highlights the heterogeneity of metastatic disease within patients and across distant sites, indicates a strong founder effect of the PT and no general niche effect, and reveals myeloid cells as the most abundant immune modulators that could be targeted at metastatic sites. We provide a unique single-cell dataset comprising primary breast tumors and their matched distant metastases, serving as a valuable resource for examining phenotypic and pathway alterations in tumor and immune cells in metastatic breast cancer.

## Materials and Methods

### Ethics statement

A written informed consent or equivalent (BASEC 2018-02282) from the patients was obtained, and the studies were conducted in accordance with recognized ethical guidelines (Declaration of Helsinki) after approval by an institutional review board.

### Clinical samples

The clinical cohort used in this study has been published and described before (19, 29); however, due to the loss of samples during handling, not all patients are part of this study. A full description of the cohort can be found in Supplementary Table S1. Briefly, the cohort was assembled from patients with breast cancer with PT and distant metastasis specimens available from the archives of the Department of Pathology and Molecular Pathology, University Hospital Zurich, in the time period of 1995 to 2019. The PT samples were classified for ER, progesterone receptor, and Her2 receptor expression and assigned a molecular subtype classification as described previously (29). The distant metastatic sites included either bone, soft tissue, liver, or brain. It cannot be excluded that patients had metastatic growths at other sites, such as lungs, monitored by imaging methods only. Four patients in this cohort were treated with preoperative chemotherapy, and the others underwent adjuvant treatment after surgery according to guidelines and available regimens at the time of the diagnosis. The cohort covers different cancer molecular subtypes, with disease-free survival (the time between the detection of PT and metastasis) ranging from 0 to 18 years. The survival data and the exact details of the treatments are not available for this cohort; only age at the PT detection and age at the metastatic lesion detection are available. Additionally, all the PT and metastatic samples have been characterized for CD8^+^ T-cell infiltration as described before (19) and classified either as immune inflamed, excluded, or desert.

### Assembly of the tissue microarray

The PT samples were from surgical specimens and metastatic samples mainly from biopsies. From each PT, two 0.6 mm in diameter tissue punches were taken from three different locations as described before (29). These locations were the intratumoral region, stroma-rich intratumoral region, and tumor margin. From the metastatic samples, an average of two 0.6 mm tissue punches, mainly from the intratumoral region, were collected except in cases in which surgical specimens were available; in those cases, samples from the stroma-rich intratumoral region and tumor margin samples were collected and annotated accordingly. The patients were randomized and samples from the clinical cohort were assembled into two tissue microarrays. Three ∼3-micron thick consecutive slices were cut from each tissue microarray and collected on microscopy slides. In total, three microscopy slides per tissue microarray were used for the IMC workflow.

### Antibody panels

Three different antibody panels were assembled with a focus on either tumor, myeloid, or T-cell phenotyping. All the panels included overlapping markers to distinguish between epithelial (panCK and E-cadherin), immune cell types (CD45, CD3, CD20, CD4, CD8, MPO, and CD68), and other stromal cells (CD31, vWF, and SMA). Iridium staining for nuclei detection and GLUT1 for general cell staining was also used in all the channels. The antibody panels were assembled based on published breast cancer IMC studies (22, 23) and further literature search. All antibodies had been validated first by immunofluorescence staining and second in the metal-conjugated form with IMC using tissue samples known to express the protein. The staining quality was evaluated based on the expected staining pattern known for each target from prior studies. Antibodies were conjugated to metals with the MaxPar Antibody Conjugation Kit (Fluidigm).

### Antibody staining

Antibody staining for all three panels was done at the same time for all six microscopy slides following a standard IMC protocol. First, slides were deparaffinized in fresh HistoClear (Biosystems) three times for 10 minutes, followed by rehydration in 100% ethanol for 2 × 5 minutes and then a graded alcohol series (ethanol:deionized water 96:4, 90:10, 80:20,70:30) for 3 minutes each. Then, heat-induced epitope retrieval was done with Tris-EDTA buffer (pH 9) at 95°C for 30 minutes in a NxGen decloaking chamber (Biocare Medical). After cooling, the slides were incubated in TBST (20 mmol/L Tris pH 7.6, 150 mmol/L NaCl, 0.1% Tween) + 3% BSA for 1 hour to reduce the unspecific antibody binding. After the blocking step, samples were stained with the respective full panel of metal-tagged antibodies and incubated overnight at 4°C (the order of panels for the consecutive cuts was as follows: tumor, T-cell, and myeloid panel). The next day, slides were washed with TBS (three times for 5 minutes) before incubating for 5 minutes with 0.5 µmol/L Cell-ID Intercalator-Ir (Fluidigm) for DNA detection. Finally, the slides were washed in TBS and dipped in deionized water before drying with pressurized air.

### Data acquisition with IMC

The IMC measurements of the tissue microarray cores were done in one batch with the Hyperion Imaging System (Fluidigm) at 400 Hz using the accompanying commercial software provided by Fluidigm. In addition to all the channels present in the panel, quality assurance channels of Ba136, Xe131, and Xe134 were acquired to check for argon gas contamination. Each 0.6 mm tissue core was acquired fully with one image per core. In rare cases when the acquisition stopped unexpectedly, the measurement was continued with a new image. In the cases in which the tissue had partially fallen off during the antigen retrieval process, the remaining tissue was acquired instead. Signal compensation slides (30) for downstream spillover compensation were also acquired as part of the data acquisition procedure.

### Data preprocessing and cell segmentation

Before further image processing, the antibody staining quality for all of the panels was visually checked using histoCAT web (https://github.com/BodenmillerGroup/histocat-web/). After that, the mcd files from the Hyperion Imaging system were processed using an established workflow in our lab available at https://github.com/BodenmillerGroup/ImcSegmentationPipeline/ (31). Briefly, Python v.3.6.6 (RRID: SCR_008394) with imctools v.2.0 (RRID: SCR_017132) available at https://github.com/bodenmillerlab/imctools was used for converting the mcd files into OME-TIFF format and generating image stacks used for single-cell segmentation. Pixel classification based on the random forest algorithm was performed using an ilastik v.1.3.3 (RRID: SCR_015246; ref. 32) together for all the images across the three panels. For training the pixel classifier for nuclear and cytoplasmic pixels, an analysis stack was assembled for a random selection of the initial images that contained a selection of channels shared across the three panels (channels for the following targets were used: HH3, SMA, panCK, CD68, GLUT1, CD3, CD11c, CD45RARO, CD8a, CAIX, CD4, CD31_vWF, E/P-Cadherin, cleaved_PARP_Caspase3, DNA1, and Ki67) and were cropped and magnified by two times (i.e., ×2). After training, the classifier was applied to the full images to obtain a set of probability maps that were then used for cell segmentation in CellProfiler v.3.19 (RRID: SCR_007358; refs. 33, 34). Pixel classification and cell segmentation for bone images were performed separately based on the same procedure. As part of the CellProfiler pipeline, the signal compensation procedure was used to correct for spillover between channels on a single-cell level (30), followed by extraction of average marker expression and spatial features, and of touching neighbors for each cell.

### Tumor–stroma masks

Tumor–stroma masks were generated using an analogous procedure to cell segmentation. A pixel classifier in ilastik v.1.3.5 was trained on the same image stack as before to distinguish between tumor cells, stroma, and background pixels guided by nuclear staining, panCK, SMA, and background pixels. After training, the classifier was applied to all the images to obtain probability maps that were then converted into tumor–stroma masks using CellProfiler v.3.19. As part of the CellProfiler pipeline, the distance to the closest tumor–stroma border was measured for each cell such that the cells under the tumor mask had a positive distance and cells under the stroma mask had a negative distance. The distances for each cell were later merged with the SingleCellExperiment object in the R environment.

### Data analysis in R

All downstream data analysis was done in R v4.0.2. First, the single-cell mean expression matrix for each panel together with the patient- and image-level metadata was converted into a corresponding SingleCellExperiment object (35). Second, cell-level quality checks (QC) were performed where all the cells with an area of fewer than 7 pixels or more than 600 pixels and images with less than 100 cells were excluded from further analysis. After QC, the T-cell–focused panel dataset included 695 images (459 from PT and 236 from metastatic sites), the myeloid-focused panel dataset included 681 images (457 from PT and 224 from metastatic sites), and the tumor-focused panel data set included 681 (454 from PT and 227 from metastatic sites).

### Clustering approaches for tumor, myeloid, and T cells

An initial clustering step to separate out the major cell types such as epithelial, endothelial, stromal, T cells, B cells, and myeloid cells was done for each panel separately using a selection of canonical cell type markers, on all cells in the dataset (i.e., from all PT and metastatic samples). For clustering, the single-cell marker expression levels were range-normalized using the 99th percentile normalization to account for outliers. An analogous clustering to Phenograph (36) was used where first a shared nearest neighbors graph based on Jaccard similarity and *k* = 5 was built using the scran R package v1.18.1 (RRID: SCR_016944; ref. 37), and second, a Louvain community detection was applied to the graph structure as implemented in igraph v1.2.6 (RRID: SCR_021238; ref. 38). The clusters were then annotated for the main cell types based on mean expression and distribution of marker expression for each cluster.

Next, for the tumor panel, all the cells from the first clustering step that were annotated as epithelial cells were pooled and clustered with the flowSOM algorithm (39) as implemented in the CATALYST package v.1.14. (RRID: SCR_017127; available from https://github.com/HelenaLC/CATALYST; ref. 40) to obtain 20 refined phenotypic clusters. For this clustering step, all the relevant tumor cell markers were used, and the marker expression values were asinh-transformed and scaled. For tumor cells, refined clustering analysis 99th-percentile normalized marker expression values were used either for graph-based clustering with *k* = 45 as described in the initial clustering step or for k-means clustering with *k* = 45 as implemented in package stats v4.0.3 (RRID: SCR_001905).

For the T-cell panel, CD8^+^ T cells and CD4^+^ T cells (including T regulatory cells) were both clustered separately using T-cell–relevant markers with the graph-based clustering approach (*k* = 50) as described in the initial clustering step. After that, based on the marker expression profile, all the clusters were manually assigned a functional metacluster annotation. T cells with no distinct expression pattern based on the measured markers were aggregated into CD4 T helper and effector CD8 T-cell subtypes.

For the myeloid panel, a few myeloid cell–specific clusters could be already annotated after the initial clustering step, such as plasmacytoid dendritic cells (DC), neutrophils, and LAMP3^+^ DC. The remaining myeloid cells were pooled and clustered again using myeloid cell–relevant markers with the graph-based clustering approach (*k* = 40) as described in the initial clustering step. For all three panels, clusters that were ambiguous were excluded from the analysis.

### Data visualization

The pixel-level images showing individual marker expressions were generated with histoCAT web (https://github.com/BodenmillerGroup/histocat-web/). Cell-type annotated segmented images were generated using the cytomapper R package (v1.2.0; ref. 41). The marker expression heatmaps, stacked bar plots, marker distribution, and principal component analysis plots using the per cell type–centered log-ratio–transformed abundance data were generated with the R packages CATALYST v1.14.1 (40) or FactoMineR v2.4 (RRID: SCR_014602). Image-level and other principal component analysis plots were generated with scater v1.18.3 (RRID: SCR_015954; ref. 42). Uniform Manifold Approximation and Projection visualizations were generated with *dittoSeq* v1.2.6 (43). Alluvial plots were generated with ggalluvial 0.12.3 and ggplot2 v3.3.5 (RRID:SCR_014601; ref. 44). All other plots were done with ggplot2 v3.3.5.

### Differential abundance analysis

Differential abundance testing was used for finding out if significant proportional differences exist for predetermined cell types between PT and metastatic sites. The test is based on negative binomial generalized linear models with quasi-likelihood functions as implemented in the EdgeR R package v3.32.0 (RRID: SCR_012802; ref. 45). Only cell types with a FDR <0.05 were reported as significant after correcting the *P* values for multiple testing with the Benjamin-Hochberg method (46). Differential abundance testing was used for tumor, immune, T-cell, and myeloid cell types. The model included the patient ID to take into account the paired design, and for immune, T-cell, and myeloid cells, the model also included the location of the sample, as metastatic sites mainly had intratumoral samples whereas PT additionally included margin and stromal-rich intratumoral regions. The same testing procedure was also applied for determining differential abundance of cell types between metastatic patient groups. In this scenario, a specific group of patients was compared with the entire remaining patient cohort, incorporating the tissue type as a covariate in the model. Results were corrected across cell types utilizing the Benjamin–Hochberg method, accounting for shared tissue area of interest across antibody panels.

### Pairwise distance and phenotypic similarity calculations

Principles of compositional data analysis were followed for comparing similarities between sites. Aitchison distance (47) was used for calculating the similarity of each patient’s PT and metastatic sample based on the proportional distribution of different cell types such as a tumor, T-cell, and myeloid cell subtypes. Aitchison distance is calculated as Euclidean distance over centered log-ratio–transformed abundances. A pseudo-count 1 was added to the frequency table before converting the frequencies into proportions and applying the centered log-ratio transformation over the proportions. For patients with multiple sites of metastasis, these were pooled for the analysis. For the tumor cell subtype, similarities over all the locations were used for PT and metastatic samples, whereas for myeloid and T cells, only intratumoral regions were used to avoid bias arising from unequal location distribution. To confirm pairwise distance calculations by the Aitchison distance, pairwise similarities were also computed for tumor cell phenotypes using the Manhattan distance (also known as Bray–Curtis similarity), employing a grid-based system for distance measurement compared with the Euclidean distance, calculating the shortest path. Also, Jaccard distance was calculated by transforming the proportions into binary values (all proportions higher than 0.5% were considered present). The Jaccard index describes the proportion of the intersection size to the union size of a set and can be converted to a distance measurement by subtracting from 1. For comparing the median distance value of the cohort with the null distribution, permutation testing was performed. To do so, patient labels of the proportion table were randomly permuted separately for the PT and metastatic samples, and each median value calculated for 1,000 iterations. To compare tumor phenotypic similarity between PT and metastases, diversity analysis was performed using the diversity function from the vegan R package v2.5.7 (RRID: SCR_011950) to compute the Shannon entropy based on tumor phenotype abundances per patient.

### PERMANOVA for evaluating the similarity of tissue sites

PERMANOVA (with 999 permutations for tumor cells and 9,999 for immune and myeloid cells) was used for comparing if the group centers based on centered log-ratio–transformed abundances differ significantly from each other (48) as applied in vegan R package. Before PERMANOVA, group dispersion differences for spatial median were determined with beta dispersion calculation with the betadispr and permutest functions from the vegan package.

### Correlation analysis of cell types across panels

Absolute numbers of endothelial and epithelial cell types, B, NK, plasma, and T-cell types, as well as myeloid cell types, were extracted from the tumor, T-cell, and myeloid panel, respectively. Subsequently, proportional frequencies of each cell type were computed at the image level, based on the summation of previously extracted cell types within a given image. Utilizing these relative cell-type frequencies, Pearson correlation coefficients were computed across the entire image set, stratified by PT and metastases.

### Cellular neighborhood analysis

Cellular neighborhood (CN) analysis was performed as proposed by Schürch and colleagues (49) and implemented in the *imcRtools* R/Bioconductor package (v1.3.6; ref. 50). For detecting CNs among epithelial cells, T cells, and myeloid cells, only images of intratumoral regions were used. For each epithelial cell, T-cell, and myeloid cell identified in the epithelial cell, T-cell, and myeloid cell dataset, respectively, the number of epithelial, T-cell, or myeloid subtypes within its 60 µm neighborhood was detected. These numbers were centered log-ratio–transformed prior to *k*-means clustering. To determine the optimal *k*, a parameter sweep for *k* between 2 and 15 was performed. The average silhouette width as calculated by the *bluster* R/Bioconductor package (v1.7.0) was used to select the optimal *k* (12 for the epithelial dataset, 14 for the T-cell dataset, and 9 for the myeloid dataset). Differential abundance testing as explained above was used to determine whether certain CNs were more or less often found in metastatic sites compared with primary tissue. The model included the patient ID to account for the paired design.

### Statistical design

Cell phenotypes were treated as proportions and calculated separately for tumor, myeloid, and T cells or for immune cells as a total. A multiple-testing correction was done with the Benjamin–Hochberg or the Bonferroni method. Nonparametric statistical tests with paired design when appropriate were used to account for non-normality in the data. The tissue microarrays were randomized at the patient level. No statistical methods were used to predetermine sample sizes and experiments, and data analysis was not performed blinded to the conditions of the experiments.

### Data availability

The data from this study are available online at https://zenodo.org/doi/10.5281/zenodo.10498366 and upon request from the corresponding author.

All code that produced the results of this study is available on Github at https://github.com/BodenmillerGroup/BCmet_publication_scripts.

## Results

### A highly multiplexed single-cell atlas of primary breast tumors and matched metastases

**Figure 1.**
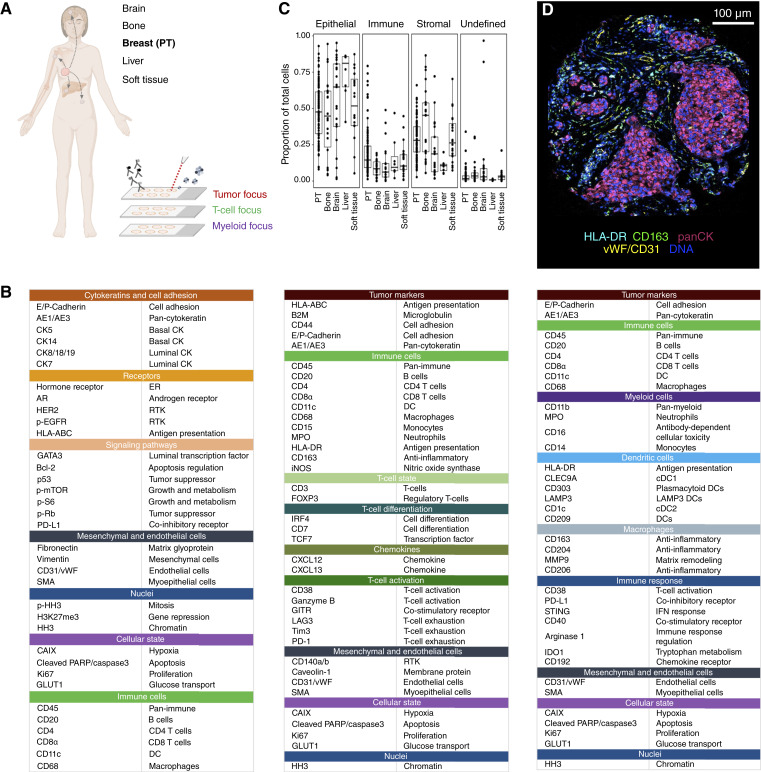
Overview of the clinical cohort and the project workflow. **A,** Overview of the clinical cohort and experimental workflow. The study includes primary breast cancer tissue samples with metastases to four different sites (bone, brain, liver, and soft tissue). Three antibody panels were used for IMC on consecutive slides, targeting tumor and immune phenotypes. **B,** Targets of the three antibody panels with their associated function. **C,** Proportion of the indicated cell types per patient in different tumor sites after the first graph-based clustering step of all cells from the tumor panel. Only images from intratumoral regions were used. Each dot represents a single patient [*n* = 528,518 cells, 464 images; *n* of patients = 87 (PT), 26 (bone), 24 (brain), 10 (liver), and 28 (soft tissue)]. **D,** Representative image of a region of interest from a PT showing expression of the indicated markers. (**A,** Created with BioRender.com.)

To identify unique features of distant metastasis, we compared FFPE PT and matched metastases from bone, brain, liver, and soft tissue in a cohort of 87 patients with breast cancer (Fig. 1A; Supplementary Tables S1 and S2). This cohort was previously characterized for B-cell and T-cell infiltration and for expression of LAG3 and PD-1 (19, 29). PT were previously categorized into luminal A (hormone receptor–positive, HER2-negative, and low Ki67), luminal B (hormone receptor–positive, HER2-variable, and high Ki67), HER2-enriched (HER2-positive and hormone receptor–negative), and TN (hormone receptor–negative and HER2-negative) subtypes.

We used highly multiplex IMC to characterize the tumor and immune cell phenotypes in these samples (22, 23, 28). We stained three consecutive cuts of each sample with three antibody panels targeting a total of 75 proteins and protein phosphorylation sites focused on either tumor cells, T cells, or myeloid cells (Fig. 1B; Supplementary Tables S3–S5). Our tumor cell panel included key clinical markers (ER, HER2, and Ki67), markers of growth-associated signaling pathways (p-mTOR, pRb, and pS6), markers relevant for immune interactions (HLA-ABC and PD-L1), and key lineage markers (CK5, CK14, and GATA3). Our two immune panels included markers that enabled phenotypic characterization of CD8^+^ and CD4^+^ T-cell subsets including regulatory (FOXP3) and exhaustion states of T cells (e.g., LAG3, PD-1, and Tim3), and to infer functional states of myeloid cells (e.g., MMP9, STING, and CD38). Many of the measured markers can already be therapeutically targeted or drugs will soon become available.

PT sections were annotated by a pathologist as intratumoral, intratumoral stromal-enriched (referred to as “stromal” hereafter), and invasive margin region, based on hematoxylin and eosin staining according to guidelines (51). The dataset included on average six images from the PT and two images from the metastatic site, per patient, per panel. In total, we recorded 2,043 images (681 images per panel) and detected more than 1 million single cells per panel. We analyzed these images using our data processing and single-cell segmentation pipelines, comparing proportional (rather than absolute) numbers of cell types due to variation in absolute cell numbers between individual images and sites (Supplementary Figs. S1–S3). To ensure against tissue-specific staining artifacts, we assessed the clustering of images and single cells on dimensionality reduction plots and checked for the expected staining patterns (Supplementary Figs. S1–S3). We highlight the need for QC of bone samples, which are particularly challenging for preparation and imaging with IMC (Supplementary Fig. S4).

We identified tumor, immune, or stromal cells using unsupervised graph-based clustering (36), followed by cluster annotation based on mean marker expression (Supplementary Figs. S5–S7). Tumor cells were the most common cell type across tissues (>50% of cells), and as expected, immune cells were the least common (<15% of cells; Fig. 1C, representative proportions, tumor panel). Metastatic sites had proportionally fewer immune cells than the PT sites. The greatest variation was in stromal cells, with liver samples having the lowest (8%) and bone the highest proportion (∼47%). A representative image of a PT sample is shown in Fig. 1D.

### Metastases share tumor cell phenotypes with paired PT but vary in composition

**Figure 2.**
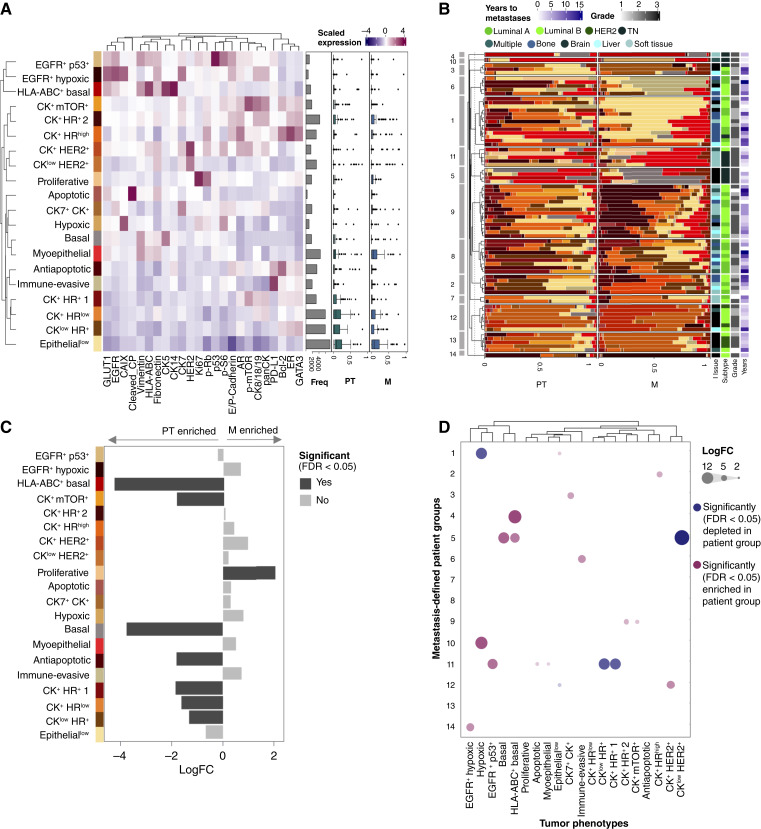
Phenotypic composition of tumor cells from PT and distant metastasis. **A,** Left, heatmap showing the median expression level for each tumor cell cluster after flowSOM clustering. Markers used for clustering are on the *x-*axis, and flowSOM clusters are on the *y-*axis. For clustering, all cells were pooled across samples (*n* = 87 patients, 559,953 cells), and the cell counts in each cluster are shown (bar plot, gray). Euclidean distance with Ward-D2 linkage was used for the hierarchical clustering of rows. Right, box plot shows the per patient proportion of tumor cell phenotypic clusters out of all tumor cells for PT (left) and metastatic (right) samples (*n* = 76 patients with paired samples). **B,** Stacked bar plot of all tumor cell phenotypic clusters as a proportion of all tumor cells in PT and metastatic samples for paired patient samples. Rows were hierarchically clustered into 14 patient groups using Euclidean distance of tumor phenotype composition in metastases with Ward-D2 linkage. **C,** Tumor cell phenotypic cluster enrichment in PT or metastasis determined with differential abundance testing using paired design. The bar plot shows the log_2_-fold abundance changes (*n* = 76 patients). **D,** Tumor cell phenotypic cluster enrichment in each metastatic patient group compared with average metastasis composition by differential abundance testing. Circles display significantly enriched (red) or decreased (blue; FDR < 0.05; multiple testing correction with the Benjamin–Hochberg method) results, with size indicating the log-transformed fold change.

To compare tumor cell phenotypes and compositions of matched PT and metastases, we first grouped all tumor cells into 20 phenotypic clusters (Fig. 2A, left). In alignment with our previous work in a different breast cancer cohort (23), we identified expected phenotypes such as CK5^+^ and CK14^+^ basal cells, apoptotic cells, Ki67^+^ proliferating cells, HER2-high cells, and various luminal cell phenotypes characterized by GATA3, varying levels of E-cadherin, cytokeratins (CK7, CK8/18/19), and hormone receptor expression. Interestingly, PT in patients with relatively early diagnosis of metastatic disease were enriched for hypoxic and EGFR^+^ tumor phenotypes (Supplementary Fig. S8). Across all patients, all 20 phenotypes were found both in primary and metastatic sites (Fig. 2A, right). For most patients, there was a high overlap in terms of the tumor cell phenotypes present at the primary and metastatic sites, but with widely varying proportions between sites (Fig. 2B). Although some phenotypes were differentially abundant between matched samples, with the proliferative Ki67^+^ pRb^+^ phenotype enriched in metastases and basal as well as luminal and antiapoptotic phenotypes enriched in PT (Fig. 2C; Supplementary Fig. S9), these phenotypes represented a very small fraction of both sample types. Thus, matched primary and metastatic samples showed the presence of similar tumor cell phenotypes, but the phenotypic proportions varied between sites.

We observed that 14 distinct patient groups could be identified by clustering the metastatic samples. Most of these groups were formed by several patients sharing similar tumor phenotype compositions, but a few were singletons, representing patients that displayed a unique profile. Most patient groups were dominated by a specific tumor cell phenotype, aligning with previous findings (23). These patient groups spanned molecular subtypes and metastatic tissue sites, and differential abundance testing identified the tumor phenotypes enriched or depleted in each group (Fig. 2D).

**Figure 3.**
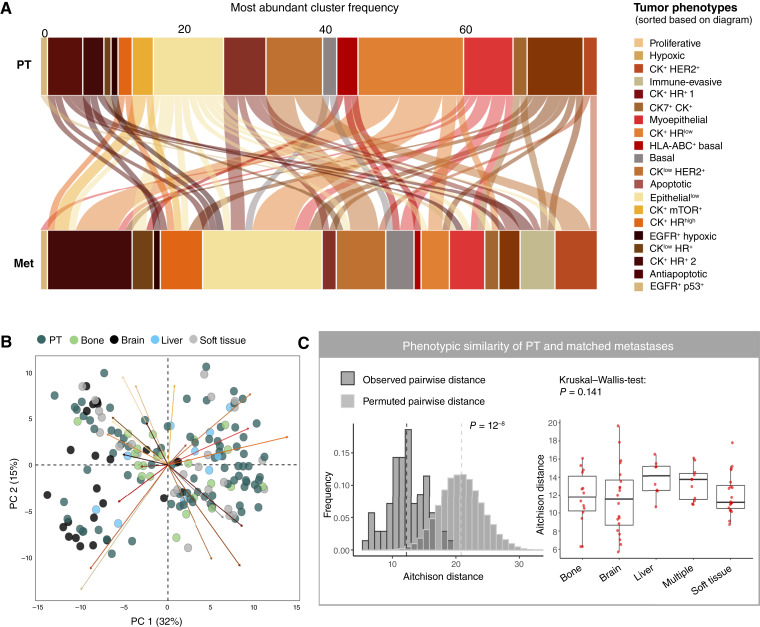
Phenotypic composition analysis of tumor cells between PT and distant metastasis. **A,** Flow chart depicting the most common phenotypic cluster in PT vs. metastatic sample. Stacked bar charts show the frequency of each cluster across the patient cohort and lines connect matched patient samples. **B,** Compositional similarity of the PT and metastatic samples displayed using PCA on the per-patient phenotypic tumor cell cluster abundances as a proportion out of all the epithelial cells (abundances were transformed with the centered log-ratio transformation). Each point represents a single patient sample of either PT or metastatic site (*n* = 76 patients). **C,** Pairwise phenotypic similarity (Aitchison distance) for the clinical cohort and randomly permuted pairwise samples, with dashed lines showing the median Aitchison distance for the patient cohort and the mean of median distances for the randomly permuted iterations (left). The right plot shows pairwise phenotypic similarity for matched samples grouped by the site of metastasis. The *P* value indicates no significant difference across all groups (Kruskal–Wallis test).

The most abundant tumor cell phenotype frequently differed between matched primary and metastatic samples (Fig. 3A; Supplementary Fig. S10A). To investigate this further, we quantitatively compared the tumor phenotype compositions of primary and metastatic sites. Overall phenotypic composition was similar in primary and metastatic samples, except for HER2-positive brain samples (Fig. 3B). Compositional comparison specifically in matched samples also showed that metastatic samples were more similar to matched rather than to random PT, although a considerable spread can be observed (Fig. 3C, left). This was further consistent with a weak positive correlation (R ranging from 0.3 to 0.6) of mean marker expression in tumor cells of matched samples (Supplementary Fig. S10B). These analyses show that patients vary in how much the phenotypic composition at the metastatic site resembles that of the matched PT, but that matched pairs are overall more similar in their composition than random nonmatched pairs.

The compositional similarity of matched metastatic and PT samples did not significantly depend on the site of metastasis (Kruskal–Wallis test; *P* = 0.141; Fig. 3C, right), which we also confirmed with other similarity measures (Supplementary Fig. S10C and S10D). Tumor molecular subtype did show small effects; however, HER2-positive and TN subtypes showed higher pairwise similarity between matched samples than luminal subtypes (Supplementary Fig. S10E). Consistent with this, tumor cell compositions analyzed separately in either PT or metastases were again similar in samples of the same molecular subtype, but not in samples with the same site of metastasis (Supplementary Fig. S11A and S11B; Supplementary Tables S6 and S7). Also, mean single-cell marker expression of tumor cells did not depend on the site of metastasis (except for brain; Supplementary Fig. S11C and S11D). PT were not more phenotypically heterogeneous than metastatic samples (Supplementary Fig. S12A), and more heterogeneous PT did not seed more heterogeneous metastasis (Supplementary Fig. S12B). The conclusions of our tumor single-cell phenotypic analysis were independent of the number of clusters we allowed (data not shown) and did not change substantially with different clustering approaches (Supplementary Fig. S12C–S12L).

In conclusion, our highly multiplexed single-cell analysis showed that tumor cell phenotypes were typically shared between matched primary and metastatic samples from patients with breast cancer, but that the proportions of phenotypes varied strongly between matched samples. Although overall tumor cell composition of matched samples was more similar than expected by chance, individual patients varied in their compositional similarity. We identified a single phenotype enriched in metastases, but none unique to metastasis, and several enriched in PT. The molecular subtype, but not the site of metastasis, was associated with the tumor cell composition in metastatic samples. Finally, the extent of phenotypic similarity between matched samples was independent of the site of metastasis, indicating an absence of site-dependent selection pressure on tumor cell phenotypes.

### A phenotypic and spatial map of the PT and metastatic immune microenvironments

**Figure 4.**
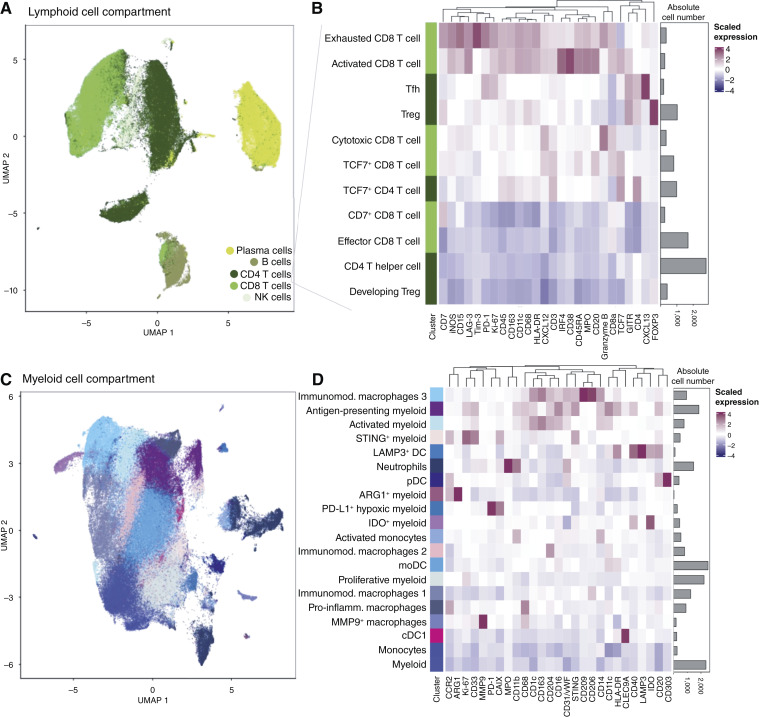
Immune cell phenotypes in PT and metastases. **A,** Uniform Manifold Approximation and Projection for Dimension Reduction (UMAP) visualization of all lymphoid cells from the T-cell panel dataset, colored by cell type (*n* = 87 patients). **B,** Heatmap showing the mean expression of T-cell–relevant markers across different T-cell phenotypic subtypes after graph-based clustering (*n* = 87 patients; *n*_cells = 91,000). Euclidean distance with average linkage was used for the hierarchical clustering of rows and columns. **C,** UMAP visualization of all myeloid cells from the myeloid cell panel dataset (*n*_cells = 161,000) excluding plasmacytoid dendritic cells (pDC), LAMP3^+^ DCs, and neutrophils, colored by phenotypic clusters (*n* = 87 patients). **D,** Heatmap showing the mean expression of myeloid-relevant markers across all myeloid cell phenotypic clusters resulting from graph-based clustering (*n* = 87 patients; *n*_cells = 161,000). Euclidean distance with average linkage was used for the hierarchical clustering of rows and columns. moDC, monocyte-derived DCs.

To enable comparison of the immune landscapes of PT and metastases, we identified broad immune cell-type categories (i.e., T cells, B cells, NK cells, myeloid, and plasma cells) using the T-cell panel dataset (Fig. 4A). We then subset all T cells, clustered the CD4^+^ T helper cells and CD8^+^ cytotoxic T cells using only T-cell–relevant markers (Supplementary Fig. S13A), and metaclustered these into functional subtypes based on similar expression profiles (Fig. 4B; Supplementary Fig. S13B). In the CD8^+^ compartment, we identified an exhausted subtype (PD-1, Tim3, LAG3, and granzyme B) and an activated subtype (CD38, GITR, and granzyme B), among others. In the CD4^+^ compartment, we observed two regulatory T-cell (Treg) subtypes, developing Tregs with lower FOXP3 expression and activated Tregs with GITR coexpression, and a subtype of T follicular helper (Tfh) cells with high CXCL13 and PD-1 expression. TCF7-high cells in both CD4^+^ and CD8^+^ compartments might represent self-renewing precursor T cells. Although our identification of these subtypes was based on manual annotation of detected markers, they align well with phenotypes identified based on genome-wide data (52), when possible to assess with shared markers.

To study myeloid cell phenotypes, we followed a similar approach. We identified myeloid cells based on expression of CD163, CD68, HLA-DR, CD11c, and CD16 (Supplementary Fig. S7A–S7C). Next, we clustered myeloid cells only based on expression levels of myeloid-relevant markers (Fig. 4C and D). We manually annotated MMP9^+^ macrophages, CLEC9A^+^ classical DC type 1 (cDC1), and various clusters of macrophages with different levels of CD204, CD163, CD209, and CD206, suggesting immunomodulatory or proinflammatory functions. Different flavors of additional myeloid cells were revealed, including a subpopulation of PD-L1^+^ hypoxic myeloid cells, immunomodulatory IDO^+^ myeloid cells, and immunosuppressive ARG1^+^ myeloid cells. Due to the heterogeneity and plasticity of myeloid cells and macrophages, we did not metacluster these cell types; we further note that any functional interpretation of marker expression may be imperfect.

Broad immune cell phenotypes could be detected at all tissue sites (Supplementary Fig. S13C). Interestingly, Tfh cells, B cells, and IDO^+^ myeloid cells were enriched in PT of patients with late-relapse, and PD-L1^+^ hypoxic myeloid cells in those of patients with early relapse (Supplementary Fig. S8). Our analysis thus provides a high-resolution compositional map of the breast cancer tumor immune microenvironment (Supplementary Fig. S13D and S13E) in matched primary and metastatic samples.

### The immune environment in metastatic tumors is tissue-dependent but affected by the PT

**Figure 5.**
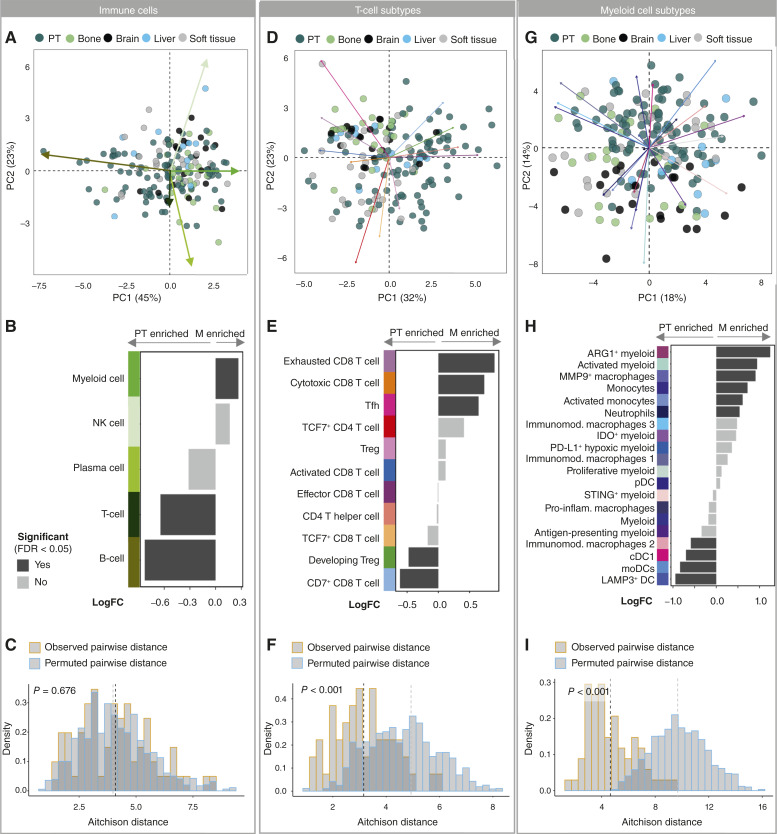
Comparison of the immune environment in PT and metastatic sites. **A,** Compositional similarity of the PT and metastatic samples displayed using PCA on the per-patient immune cell abundances (*n* = 76 patients; after applying the centered log-ratio transformation). Each point represents a single patient sample of either PT or metastatic (M) site. **B,** Immune cell type enrichment in PT or metastasis determined via differential abundance testing using paired design. Bar plot shows the log_2_-fold abundance changes (*n* = 76 patients). **C,** Compositional similarity between each individual patient-matched PT and metastatic sample based on proportional abundance of simple immune composition. The similarity was determined with Aitchison distance. Distribution of median Aitchison distance calculated over all the samples after every random permutation (*n* = 1,000 iterations) of sample labels. *P* value was calculated as the probability of observing a permutation median lower than the true patient cohort median. The dashed lines show the median Aitchison distance for the patient cohort and the mean of median distances for the randomly permuted iterations. **D**–**I,** As in **A–C,** for T-cell subtypes (**D–F**) and myeloid cell subsets (**G–I**). moDC, monocyte-derived DCs; pDC, plasmacytoid dendritic cells.

We next compared the composition of the immune microenvironment in PT and metastatic samples. We compared only the intratumoral regions as metastatic samples were mainly intratumoral, and there were immune compositional differences between the margin and intratumoral regions of PT (Supplementary Fig. S13F). PT were similar to metastatic samples in their broad immune cell-type abundances (Fig. 5A). Paired differential abundance testing however showed an enrichment of B cells and T cells in PT and an enrichment of myeloid cells at metastatic sites (Fig. 5B), reflecting stable myeloid but reduced lymphoid cell densities at metastases relative to PT (Supplementary Fig. S14A). Metastatic samples were not more similar to their matched PT than to random PT in their overall immune cell composition (Fig. 5C). Primary and metastatic tumors differed in T-cell subtype abundance (Fig. 5D), driven by a higher proportion of antigen-experienced phenotypes (exhausted CD8 T cells and Tfh cells) and of cytotoxic CD8^+^ T cells in metastases relative to matched PT, and by lower proportions of CD7^+^ CD8 T cells and developing Treg phenotypes (Fig. 5E), consistent also with cell density analyses (Supplementary Fig. S14B). In contrast to overall immune composition, matched samples were more similar in their T-cell subtypes than a random comparison (Fig. 5F). Similarly, primary and metastatic sites grouped separately based on the myeloid cell phenotypic cluster abundances (Fig. 5G). This was driven by an enrichment of the proportions of ARG1^+^ myeloid cells, MMP9^+^ macrophages, and neutrophils, among others, in metastatic samples, whereas PT were enriched in LAMP3^+^ DCs, the CD204high/CD163low immunomodulatory macrophages, and cDC1s (Fig. 5H); again, cell densities of myeloid cell subtypes showed similar trends (Supplementary Fig. S14C). As for T-cell subtypes, matched samples were also more similar in their myeloid cell phenotypic clusters than expected at random (Fig. 5I).

**Figure 6.**
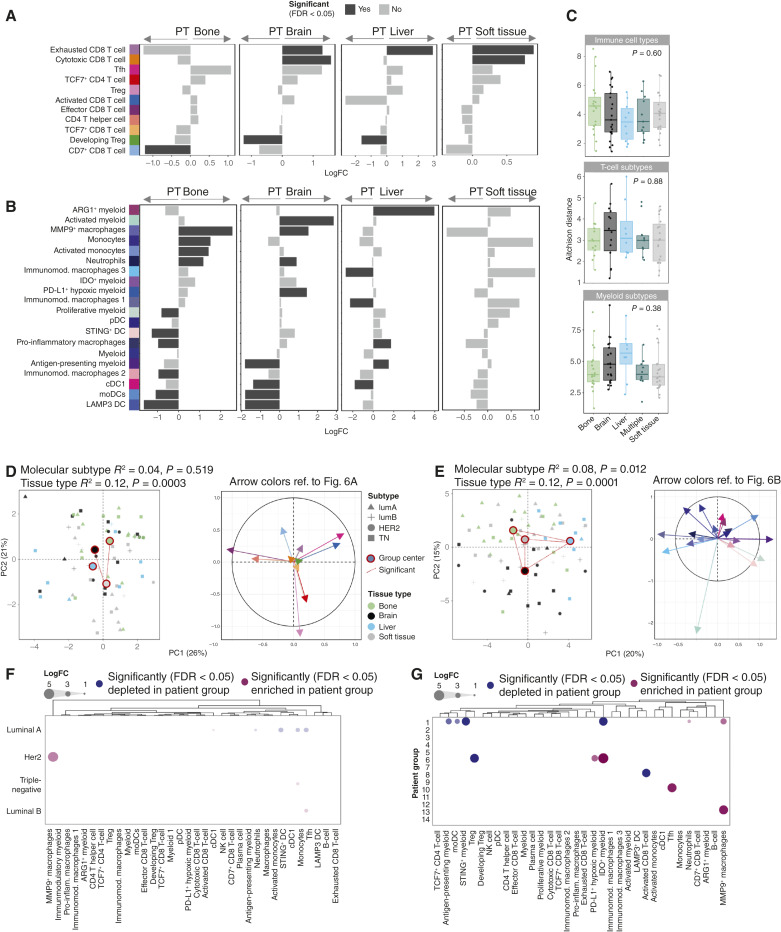
Immune environment in different metastatic sites and the effect of the PT on the immune cell composition. **A** and **B,** T-cell (**A**) and myeloid cell (**B**) subtype enrichment in PT or metastasis determined via differential abundance testing using paired design and grouped by the site of metastasis. Bar plot shows the log_2_-fold abundance changes (*n* = 76 patients). **C,** Boxplot of compositional similarity between each individual patient-matched PT and metastatic sample based on the proportional abundance of cell types grouped by the site of metastasis for general immune cell types (top), T-cell subtype proportions (middle), or myeloid cluster proportions (bottom). Plotted is the Aitchison distance as a measure of compositional dissimilarity. **D** and **E,** Compositional similarity of metastatic samples displayed using PCA on the per-patient immune cell type abundances (*n* = 76 patients; after applying the centered log ratio transformation) and corresponding variable factor map [right; only factors with a combined absolute contribution (to PC1 + PC2) >4 are displayed]. T-cell subtypes (**D**) and myeloid cell subtypes (**E**) are visually represented using arrow colors corresponding to the cell types listed in **A** and **B**, respectively. Each point represents a single patient sample. Colored squares indicate group centroids. Results of the PERMANOVA test for centroid difference are given (top). Patients with multiple metastatic sites were excluded to ensure the independence of individual samples. **F** and **G,** Immune cell phenotypic cluster enrichment in each clinical breast cancer subtype (**F**) or metastasis-defined patient group (**G**) at metastatic sites compared with average metastasis composition by differential abundance testing. Circles display significantly enriched (red) or decreased (blue; FDR < 0.05; multiple testing correction with the Benjamin–Hochberg method) results, with size indicating the log-transformed fold change.  moDC, monocyte-derived DCs; pDC, plasmacytoid dendritic cells.

We further asked if there were tissue-dependent signatures of the metastatic immune microenvironment. Differential abundance testing between primary and metastatic tumors showed few differences in T-cell subtypes between the sites (Fig. 6A). Exhausted CD8 T cells were enriched in brain, liver, and soft tissue samples, whereas bone samples showed depletion in CD7^+^ CD8^+^ T cells. Additionally, brain and soft tissue samples had a higher proportion of cytotoxic CD8^+^ T cells, and PT that metastasized to liver and brain had a higher proportion of developing Tregs. The proportional increase in cytotoxic and exhausted CD8 T cells in brain metastases occurred despite an overall reduction in T-cell density in these samples (Supplementary Fig. S14D), consistent with trends toward lower density of most other T-cell subtypes in brain metastases (Supplementary Fig. S14E). There was also a strong reduction in density of most T-cell types in bone metastases relative to PT (Supplementary Fig. S14E).

Myeloid phenotypic clusters showed enrichment of many different phenotypic clusters at different metastatic sites (Fig. 6B), for example, enrichment of the MMP9^+^ macrophages in bone and brain samples, neutrophils, and PD-L1^+^ hypoxic myeloid cells in brain samples, whereas corresponding PT were enriched in LAMP3^+^ DCs, monocyte-derived DCs, and cDC1s. The only exception was the soft tissue samples where no myeloid cluster showed a significant difference between the matched metastatic and PT samples. These proportional differences were consistent with trends in myeloid cell subtype densities at different sites (Supplementary Fig. S14F). The compositional similarity between PT and metastatic samples did not depend on the metastatic tissue site either for general immune cell types, T-cell subtypes, or myeloid clusters (Fig. 6C).

Finally, we probed immune signatures specifically in metastatic tumors. T-cell composition differed significantly between tissue sites, but with group centers located close together (Fig. 6D, left). These differences were driven by the variations between soft tissue and either brain or bone samples and explained mainly by TCF7^+^ CD4 T cells and Tregs (Fig. 6D, right). Myeloid cells in contrast showed strong tissue-dependent compositional signatures (Fig. 6E), with significant differences between all the tissue sites (Supplementary Tables S8 and S9). Immune cell composition also diverged significantly between molecular subtypes for the myeloid but not the T-cell compartment (Supplementary Fig. S6D and S6E; Supplementary Tables S8 and S9). Furthermore, in pairwise comparisons of immune cell phenotype abundance between metastases of each molecular subtype and the average abundance across all metastases (Fig. 6F), luminal A metastases showed a significantly reduced presence of Tfh, monocytes, STING^+^ DCs, and neutrophils compared with the other subtypes. In contrast, the Her2 subtype was associated with MMP9^+^ macrophages. Although these results should be interpreted with caution because the numbers of patients within each molecular subtype group are small, they are in agreement with prior studies of PT (21, 53). We also observed differences in the metastatic immune microenvironments depending on the tumor phenotypic composition (Fig. 6G) of our defined patient groups (Fig. 2B). Of particular interest, patient group 6, enriched for immune-evasive tumor phenotypes, was also significantly enriched for PD-L1 hypoxic and IDO^+^ myeloid cells and depleted of Tregs.

In conclusion, T-cell abundance was reduced, but the relative proportion of Tfh and exhausted CD8^+^ T cells was increased at metastatic sites relative to matched PT. The myeloid cell compartment showed strong tissue-specific compositional signatures, with several different macrophage phenotypes enriched at different metastatic tissue sites. Similarly, molecular subtypes were associated with differential abundance of DC phenotypes, Tfh, neutrophils, and MMP9^+^ macrophages.

### PT and metastases share spatial tumor and immune CNs

**Figure 7.**
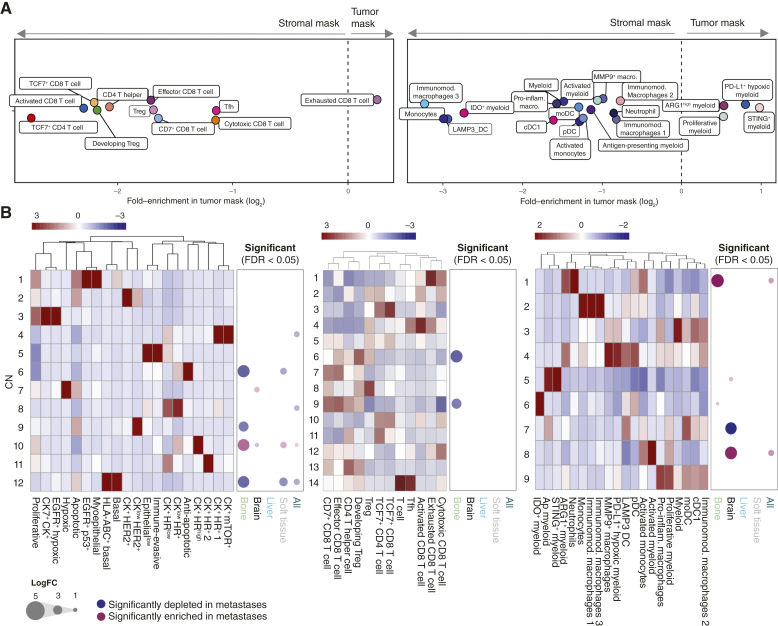
Spatial T and myeloid CNs are shared between primary tissue and metastatic sites. **A,** Relative enrichment of T-cell (top) and myeloid (bottom) subtypes in tumor vs. stromal patches across the whole cohort (*n* = 87). **B,** Heatmaps illustrating cell-type frequencies per tumor (left), T-cell (middle), or myeloid cell (right) neighborhood. Columns are *z*-scored for visualization purposes. The corresponding bubble diagrams show significant results (FDR < 0.05) of differential abundance tests comparing the CNs in metastases to those in PT. Diameter reflects the log_2_-fold change in abundance (*n* = 76). moDC, monocyte-derived DCs; pDC, plasmacytoid dendritic cells.

Finally, we spatially characterized immune cell subtypes within primary and metastatic tumors. First, we calculated the relative enrichment of T-cell and myeloid cell subtypes within tumor and stromal masks within each image across the cohort. The only T-cell subtype enriched within tumor masks was CD8^+^ exhausted T cells, as expected because chronic T-cell receptor engagement leads to T-cell exhaustion (Fig. 7A, left; refs. 54, 55). Only a few myeloid clusters were enriched in tumor masks, including PD-L1^+^ hypoxic myeloid cells and STING^+^ myeloid cells (Fig. 7A, right). Myeloid cell subtypes with the highest enrichment in stromal masks were also enriched in margin images (Supplementary Fig. S13F).

Second, we conducted a spatial analysis of all cell types across all images (i.e., generated using all antibody panels). We used the few markers in the tumor panel that identified key cell types also in the immune and stromal compartments to test for changes in interaction between PT and metastases. We observed subtle differences, for instance, T cells showed significantly greater avoidance of antiapoptotic tumor cells in PT compared with metastases (Supplementary Fig. S15A), but overall, changes were limited. Next, we tested for significantly higher or lower correlation of cell-type abundance per region of interest. Here we asked whether cell types identified within each panel were correlated with other cell types in sequential images stained with the other two panels. Because the analyzed data are from sequential tissue sections, correlations report on spatial relationships on a length scale corresponding to the image dimensions (500 μm × 500 μm). These analyses showed few striking differences between primary and metastatic tumor correlations, and we detected changes mainly between different immune cell types (Supplementary Fig. S15B and S15C). Of note, p53^+^ EGFR^+^ tumor cells displayed high correlation (i.e., occupied a shared region) with different T-cell subtypes in PT but not in metastatic sites, whereas many T-cell subsets occupied a shared region with ARG1^+^ myeloid cells in metastases but not PT.

Third, we asked if metastatic sites harbored spatial niches or local enrichments of cell types, also referred to as CN (49), and if these were different in metastasis versus PT. Because tumor cells, T cells, and myeloid cells were analyzed by staining consecutive sections, we conducted the CN analysis separately for each of these (Supplementary Fig. S16A–S16C; Materials and Methods). We identified 12 tumor CNs, e.g., CN3 with proliferative, EGFR^+^ hypoxic, and CK7^+^ tumor cells, CN7 consisting of hypoxic cells, and CN8 comprising immune-evasive and epithelial-low phenotypes (Fig. 7B, left; Supplementary Fig. S16D and S16F). Hormone receptor–high CN10 was enriched at multiple metastatic sites relative to the PT, except for the brain and liver. Brain metastases were depleted for CN10 and enriched for hypoxic CN7 instead. Apart from the brain, we observed few site-specific patterns in epithelial CNs.

Our analysis detected 14 T-cell CNs (Fig. 7B, middle), each containing unique combinations of T-cell subtypes, e.g., exhausted CD8 and GZMB-producing CD8^+^ T cells in CN1, TCF7-high CD4^+^ and CD8^+^ T cells in CN3 and activated CD8^+^ T cells in CN4. Comparing the abundance of each T-cell CN between primary and metastatic sites revealed few differences (Fig. 7B, middle). The exceptions were CN6 (containing mainly developing Tregs) and CN9 (CD8^+^ effector and CD8^+^/CD7^+^ T cells), which were depleted in bone metastases relative to PT (Fig. 7C, middle); Supplementary Fig. S16D–S16F]. Overall, the spatial architecture of T cells was similar between the primary and metastatic tumors.

We identified nine myeloid cell CNs (Fig. 7B, right; Supplementary Fig. S16B), with brain metastases being depleted for CN7, containing DCs, and enriched for CN8, containing predominantly activated myeloid cells (Fig. 7B, right; Supplementary Fig. S16D–S16F). These results are in line with the differential abundance of these phenotypes in brain metastases versus PT (Fig. 6B), showing that these phenotypes not only changed abundance but form spatial CNs that also differed in abundance between these samples. We also observed that bone metastases were enriched for CN1, containing neutrophils and ARG1^+^ myeloid cells, compared with primary tissue. Taken together, our spatial analyses of primary and distant metastatic tumors show an overall similar spatial organization at both tumor sites, but with differences in the abundance of specific local neighborhoods.

## Discussion

Metastatic breast cancer is rarely diagnosed concomitantly with the PT, but more typically after treatment of the primary disease. The duration from primary diagnosis to metastasis can vary widely, at 1 to 5 years for HER2^+^ and TN subtypes and up to 20 years or more for luminal disease (56). Current knowledge of the metastatic tumor ecosystem is limited, despite its importance for effective disease treatment. Although previous studies have identified changes in cell populations in lymph node metastases relative to PT (27, 57), there is to date no large-scale single-cell protein-level comparison of primary breast tumors and matched distant metastases. We have addressed this problem by conducting a deep phenotypic characterization of tumor and immune cell types in matched primary and metastatic disease. This allowed us to identify shared and distinct characteristics of primary and metastatic tumors, evaluate the impact of metastatic site or molecular subtype on cellular phenotypes, explore the role of the tumor immune microenvironment on tumor phenotypes, and identify potential therapeutic targets for improved disease management.

We used IMC to analyze 75 unique antibody targets in matched primary breast tumors and distant metastatic sites from 87 individuals. The cohort encompassed all major breast cancer metastatic sites, except for the lung, and covered all breast cancer subtypes. We found that tumor cell phenotypes were shared between matched PT and metastases, but that the phenotypic composition varied, depending on the patient. Furthermore, this patient-specific variation was independent of the metastatic site but affected by the molecular subtype. Selective pressure exerted by endocrine therapies could potentially explain the greater divergence in metastatic versus primary luminal tumors than in HER2 and TN subtypes.

We recently described a similar analysis in primary breast tumors and their matched lymph node metastases, in a cohort comprising 205 individuals (27). As we saw here for distant metastases, this previous work showed that matched primary and lymph node metastases also differ in tumor cell phenotypic composition, although again the same phenotypes could typically be detected in both samples of a matched pair. These similarities between matched pairs are much more unexpected for distant metastases. In contrast to lymph node metastases, which are typically detected at the same time as the PT, distant metastases may be detected years or decades later, and at more distant sites in the body. This suggests a strong founder effect of the PT. Our results require validation in an independent cohort. Nevertheless, these results suggest that minor cell populations within a primary breast tumor may affect the risk of metastasis and should therefore be evaluated for prognostic effects.

We note that analyses of three independent cohorts of patients with breast cancer with tumor-focused antibody panels (23, 27) have consistently identified similar tumor cell phenotypes and single-cell pathology groups (i.e., compositions of tumor cell phenotypes), defined in PT and shown to be prognostic in breast cancer (23). This increases confidence that these phenotypes are characteristic of the disease.

A limited number of phenotypic markers, as in this study, cannot conclusively infer mechanisms of metastasis and tumor evolutionary paths. Nevertheless, our data fit a linear model in which cells capable of establishing metastatic sites evolve within the PT before dissemination. In this scenario, the tumor cells at metastatic sites should resemble those of the PT, as we observed. The alternative parallel model proposes that tumor cells leave the PT early but undergo further mutations and progression to acquire a phenotype necessary for metastatic growth (58); this should result in distinct phenotypes between PT and metastases. Within the limitations of our antibody panel, we did not observe unique metastatic phenotypes. Instead, we found the same tumor cell phenotypes in PT and matched metastases, albeit at varying frequencies. A recent study that employed whole-genome sequencing to compare breast cancer metastases to PT also demonstrated strong similarity in single mutations (59). Our study also informs on the “seed and soil” hypothesis (60), which proposes that metastases form in organs with tumor cell–compatible microenvironments, implying an influence of the metastatic site on the composition of the metastatic tumor. However, our results show that the composition of the PT, rather than the distant tissue site, is the main determinant of metastatic composition. Additionally, we observe strong intrasite tumor heterogeneity, with the exception of brain metastasis, further arguing against strongly selective properties of the metastatic sites. These interpretations should be considered speculative and should form the basis for future studies.

Our findings have implications for understanding whether the immune escape strategies of metastases recapitulate those of the PT or are shaped by the secondary site. Although we detected a higher proportion of exhausted and cytotoxic T cells in metastatic compared with matched PT, as well as a site-specific enrichment of macrophages and of putative matrix remodeling phenotypes, the similarity in the immune cell types between matched samples exceeded that expected by chance. This suggests a complex interplay between environmental and tumor intrinsic features in shaping the composition of the T-cell and the myeloid cell compartments at both locations. The PT may influence the immune cell composition of temporally proximal metastases through mechanisms such as initial immune cell priming or long-distance effects mediated by secreted factors, potentially shaping the immune landscape of distant sites in a manner similar to its own immune profile (61, 62). Furthermore, the increased abundance of exhausted and cytotoxic T cells in metastases, as also seen previously (19), may have therapeutic implications because the presence of cells with cytotoxic potential in metastatic samples may identify patients who would benefit from immunotherapy (63–65).

Several myeloid cell types were enriched in metastatic versus matched primary samples in this cohort. Macrophages play diverse roles in shaping the breast tumor microenvironment, exhibiting spatial and tissue-specific effects (66, 67). Studies across various cancers indicate a spectrum of heterogeneity among tumor-associated macrophages (TAM), with some cells exhibiting antitumoral properties by promoting inflammation and cytotoxicity and others being protumoral activities, supporting angiogenesis and immune evasion (68–70). We observed an MMP9^+^ subtype enriched in metastasis, suggesting increased matrix remodeling capacity at the metastatic site. We also observed an enrichment in CD163^+^/CD204^+^/CD206low immunomodulatory macrophages whose role in breast cancer metastasis has not been studied. CD163 and CD204 are general markers for macrophage activation in human tumors, and CD163^+^/CD204^+^ TAMs promote T-cell apoptosis and immunosuppression in oral squamous cell carcinoma (71). In parallel, we observed lower proportions of LAMP3^+^ DCs and cDC1s in metastatic samples compared with the matched PT. Immune escape is a prerequisite for successful metastatic outgrowth, and our study suggests that a high myeloid:DC ratio and an increase in immunosuppressive TAM phenotypes might be contributing factors. Notably, myeloid cell composition varied across different metastatic tissue sites with a few unique tissue-specific spatial myeloid neighborhoods. The liver, the primary site of the urea cycle, showed an increase in ARG1^+^ myeloid cells, which play a crucial role in both the urea cycle and immunoregulation. This supports the notion that metastasis-associated myeloid cells often arise from tissue-resident cells and are thus likely to display tissue-specific characteristics, whereas T cells are more often recruited to the metastatic site (72). Furthermore, these findings suggest that different mechanisms of immunosuppression may be active in different tissues.

Studies of human metastasis samples face inherent challenges. First, the substantial interpatient heterogeneity we observed in how well each metastatic tumor reflected its matched PT in phenotypic composition may be due in part to effects of treatment, which has been shown to affect clonal similarity of matched primary and metastatic sites (73) as well as similarity of hormone receptor and HER2 expression (14, 15). The heterogeneity could also be partly caused by new mutations acquired during disease progression (14, 59). We could not however directly investigate such effects because our cohort lacks information on patient treatment and mutation profiles. Second, our study has a limited number of regions from metastatic samples, thus some patient-specific differences could be due to sampling. We mitigated this effect by thoroughly sampling the PT from different tumor locations. Additionally, refined tissue-specific effects could be uncovered with larger patient numbers and with additional tissue-specific markers, for example, markers for microglia, which might be abundant in brain metastases but could not be identified with our antibody panel. In general, our panels were designed to focus on characterizing the tumor cell phenotypes using common clinically relevant markers and on markers of the functional states of myeloid cells. Third, our findings will require validation in independent cohorts.

In summary, we examined the tumor and immune cell phenotypic composition in matched primary and distant metastatic breast tumor samples in unprecedented detail and at single-cell resolution. We demonstrated the diversity of metastatic disease among patients and identified myeloid cells as the predominant immune modulators at metastatic sites, indicating that they may be a potential target for metastasis specific therapy. Our dataset provides a unique resource to compare primary and metastatic tumors of breast cancer.

## Supplementary Material

Supplementary Tables Supplementary Tables 1-9

Supplementary Figures Supplementary Figures 1-16
