# Supplementary material for: Distant Metastases of Breast Cancer Resemble Primary Tumors in Cancer Cell Composition but Differ in Immune Cell Phenotypes
Source: Cancer Res. 2024 Oct 22;85(1):15–31. doi: 10.1158/0008-5472.CAN-24-1211 (PMC11694063; doi:10.1158/0008-5472.CAN-24-1211)
Supplement: Supplementary Figures — 1-16 [file can-24-1211_supplementary_figures_suppsf1-16.pdf]

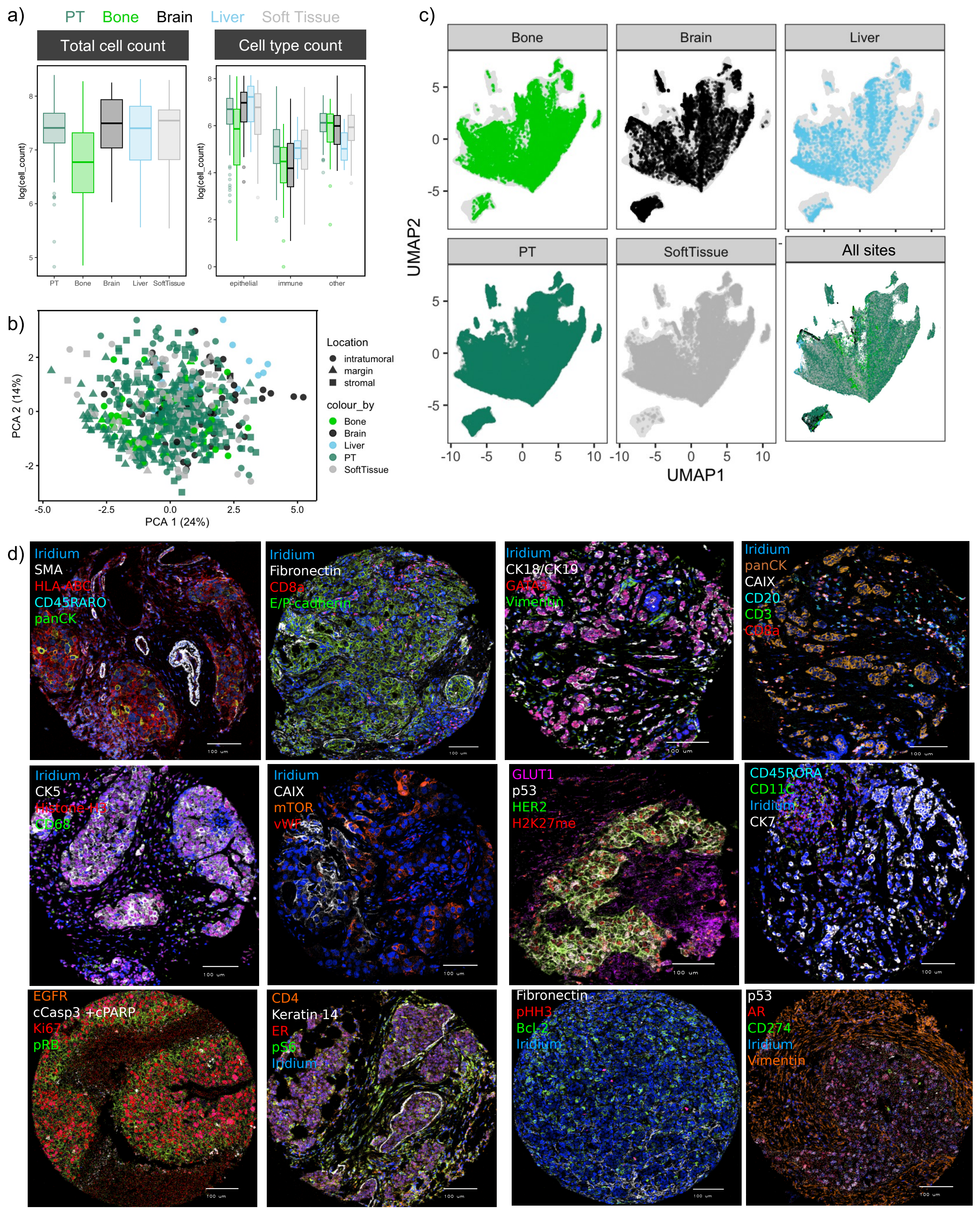

Supplementary figure 1. Quality assessment of images acquired with the tumor panel. a) Box plots display the absolute number of cells either overall (left) or in each class (epithelial, immune, other; right) in the primary tumor and the indicated metastatic location. Data are shown per intratumoral image. b) PCA of image-level arcsinh-transformed mean marker expression (n = 87 patients, 681 images). All markers were used except nuclear markers DNA1, DNA2, and HH3 to avoid nuclear staining-dependent similarity between images. c) Single-cell level UMAP calculated over a subset of markers (SMA, panCK, Vimentin, CK8/18/19, CD68, CK14, CD20, CD3, CD11c, HER2, CK5, GATA3, CK7, CD8a, Fibronectin, CD4, CD31_vWF, E/P-Cadherin) using 99th percentile range normalized intensity values. In images with <300 cells, all cells were used, otherwise 300 cells were randomly sampled. Cells are colored by tissue of origin (n = 87 patients, n_cells = 198,380 cells, 681 ROIs). d) Representative images of sections stained with the tumor panel, with each marker represented at least once.

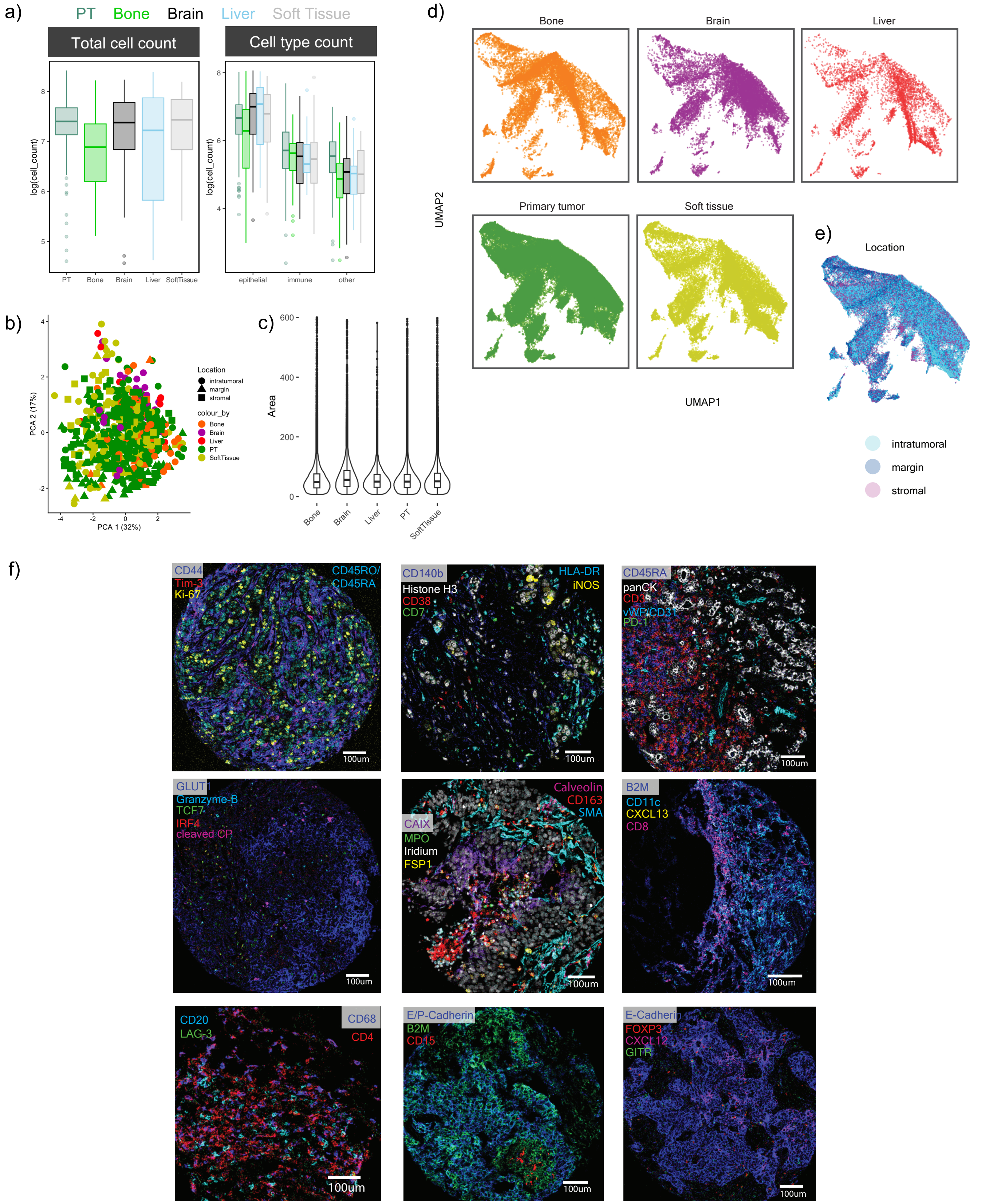

Supplementary figure 2. Quality control of images acquired with the T cell panel. a) Box plots display the absolute number of cells either overall (left) or in each class (epithelial, immune, other; right) in the primary tumor and the indicated metastatic location. Data are shown per intratumoral image. b) PCA of image-level arcsinh-transformed mean marker expression (n = 87 patients, 695 images). All the markers were used except nuclear markers of DNA1, DNA2, and HH3 to avoid nuclear-staining dependent similarity between images. c) Average cell size in pixels (1 pixel = 1μm^2) across tissue types (695 images, n = 87 patients). d)-e)) Single-cell level UMAP calculated over a subset of markers (CD140b, SMA, Caveolin-1, CD31/vWF, CD4, CD3, HLA-DR, CD11c, CD68, CD163, CD7, FOXP3, CD38, CD8a, CD20, MPO, CD15, E/P-Cadherin, panCK) using 99th percentile range normalized intensity values. From images with <300 cells all cells were used, otherwise 300 cells were randomly sampled. Cells are colored by (d) tissue of origin (n = 87 patients, n_cells = 199,822, total of 695 ROIs) or (e) location of acquisition. f) Representative images from the immune panel with each marker represented at least once.

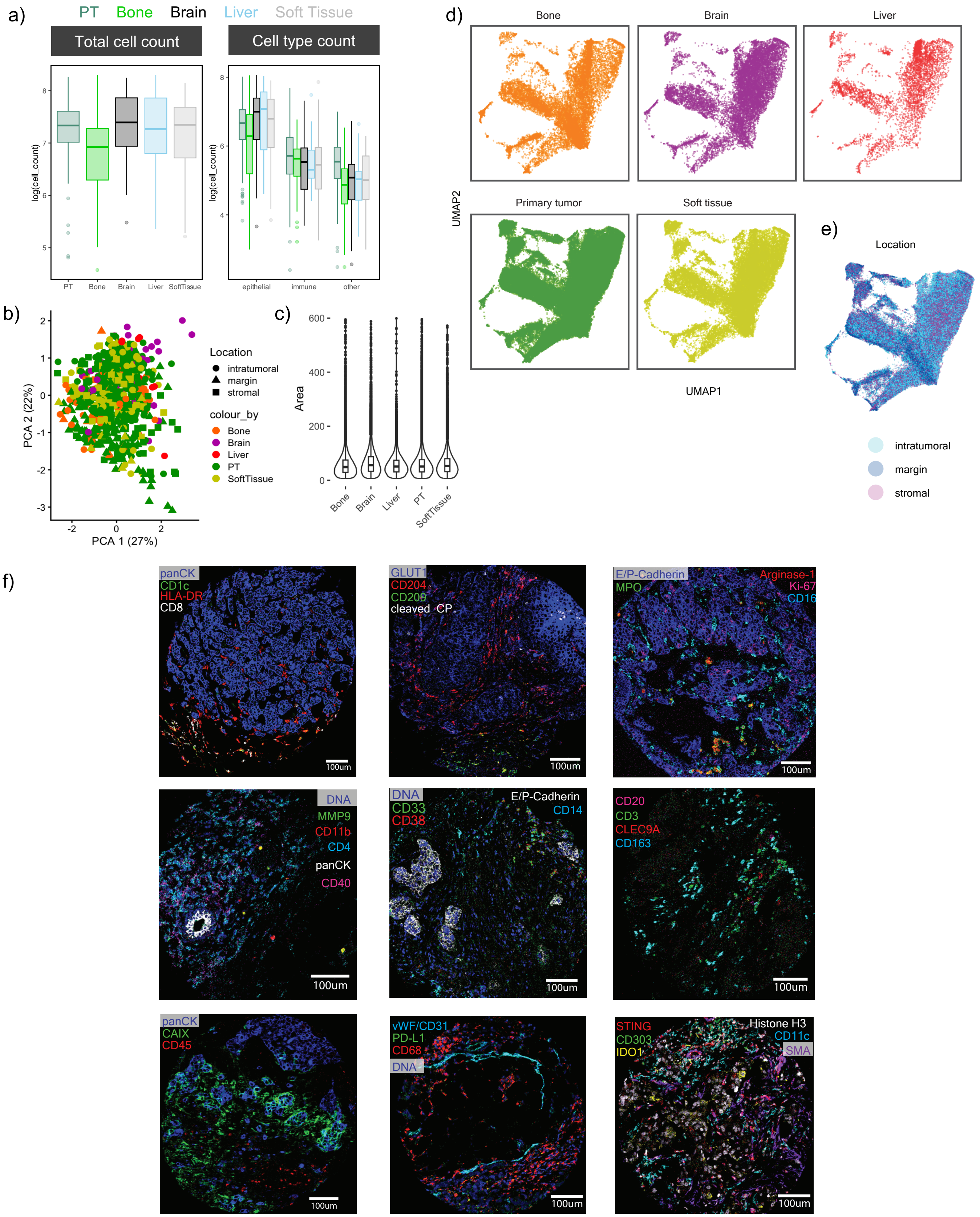

Supplementary figure 3. Quality control of images acquired with the myeloid cell panel. a) Box plots display the absolute number of cells either overall (left) or in each class (epithelial, immune, other; right) in the primary tumor and the indicated metastatic location. Data are shown per intratumoral image. b) PCA of image-level arcsinh-transformed mean marker expression (n = 87 patients, 681 images). All the markers were used except nuclear markers of DNA1, DNA2, and HH3 to avoid nuclear-staining dependent similarity between images. c) Average cell size in pixels (1 pixel = 1μm^2) across tissue types (681 images, n = 87 patients). d)-e) Single-cell level UMAP calculated over a subset of markers (CD4, CD303, CD3, CD8a, CD14, CD11c, CD16, CD68, CD163, HLA-DR, LAMP3, CD38, CD20, MPO, CD31/vWF, E/P-Cadherin, SMA, panCK) using 99th percentile range normalized intensity values. From images with <300 cells all cells were used, otherwise, 300 cells were randomly sampled. Cells are colored by (d) tissue of origin (n = 87 patients, n_cells = 196,734, total of 681 ROIs) or (e) location of acquisition. f) Representative images from the myeloid panel with each marker represented at least once.

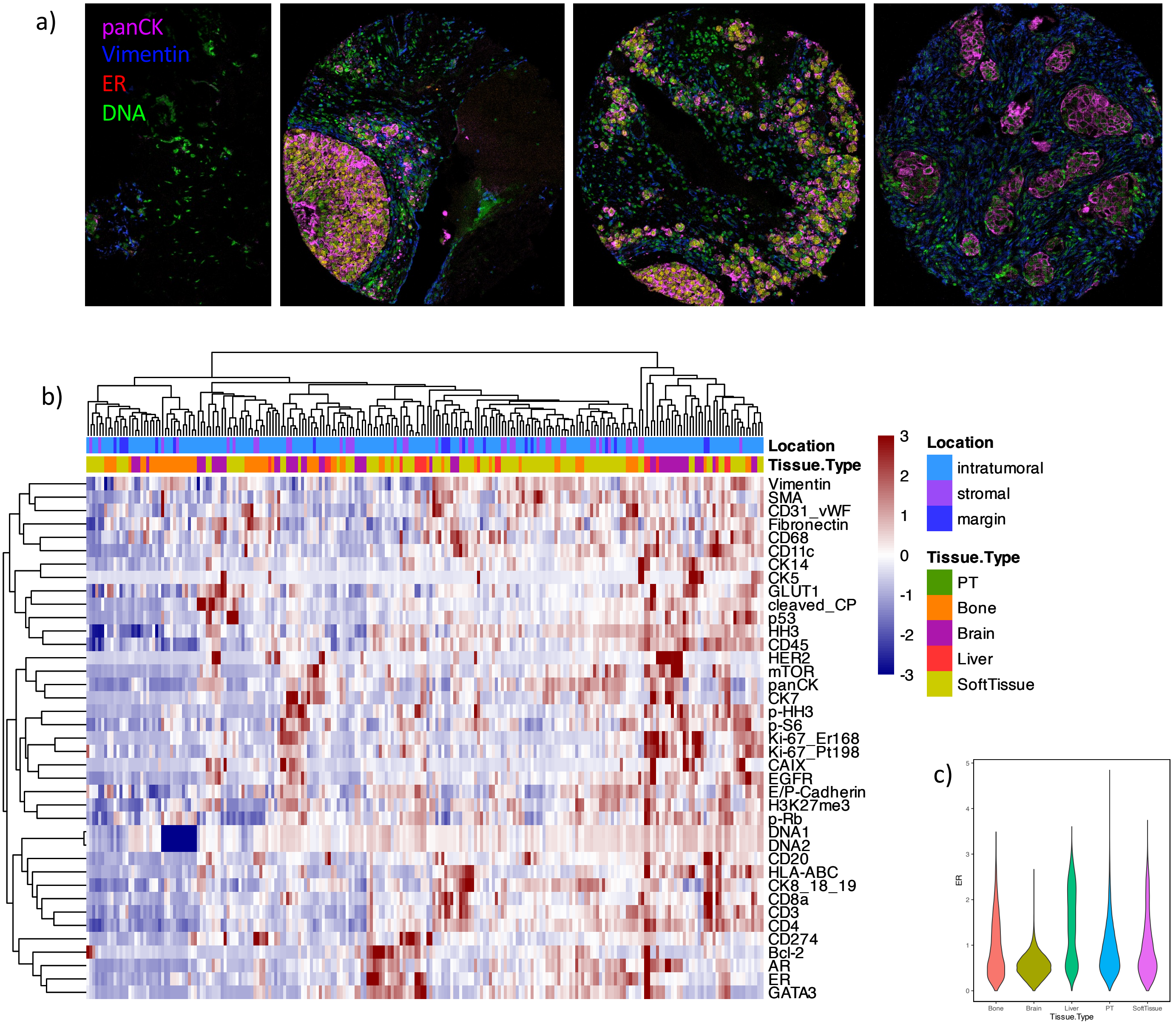

Supplementary figure 4. Quality control for breast cancer metastases in bone samples. a) Exemplary images from bone metastases. Examples include images of low quality (left), lower cell count due to anatomical structures (middle left), as well as staining examples for estrogen receptors from a ER+ (middle right) and ER- patient (right). b) Clustering of images at metastatic sites based on their mean marker expression. Annotations indicate their location (intratumoral, stromal, margin) as well as their tissue site (PT, bone, liver, brain, soft tissue). c) Single-cell expression levels of estrogen receptor (ER) of epithelial cells in different tissue types.

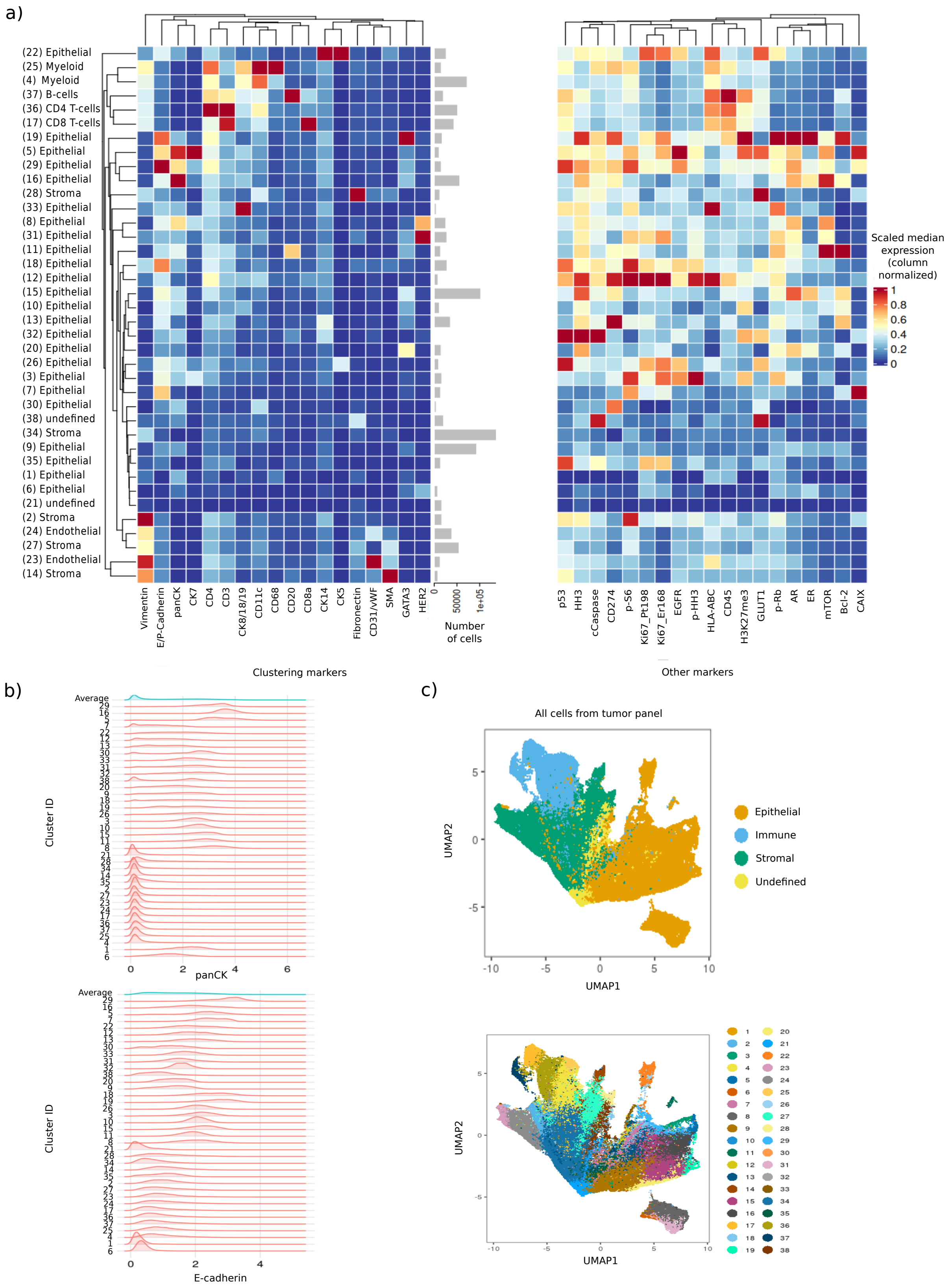

Supplementary figure 5. Initial clustering of single-cell data from tumor panel to identify epithelial, immune, and stromal cells. a) Heatmap of cluster-level mean expression. Graph-based clustering with Louvain community detection (k=5, jaccard) was used over all the cells (n = 87 patients, n_cells = 1,082,825, total of 681 images). Only a subset of key markers was used for clustering to identify epithelial, immune, and stromal cells (SMA, panCK, Vimentin, CK8_18_19, CD68, CK14, CD20, CD3, CD11c, HER2, CK5, GATA3, CK7, CD8a, Fibronectin, CD4, CD31_vWF, E/P-Cadherin). Clusters were calculated over the 99th percentile range-normalized values. Middle: Bar chart with the number of cells in each cluster. Right: Heatmap of cluster-level mean expression for markers not used for clustering. b) Distribution of arcsinh-transformed expression for panCK and E-cadherin for all the clusters. c) Single-cell level UMAP calculated over the same markers as used for clustering using the 99th percentile range normalized intensity values. From images with <300 cells all cells were used, otherwise 300 cells were randomly sampled. Cell type annotations are as in (a) (n = 87 patients, n_cells= 198 380, 681 images). d) Same as (c) with cells colored by cluster ID. Cluster annotations are as in (a).

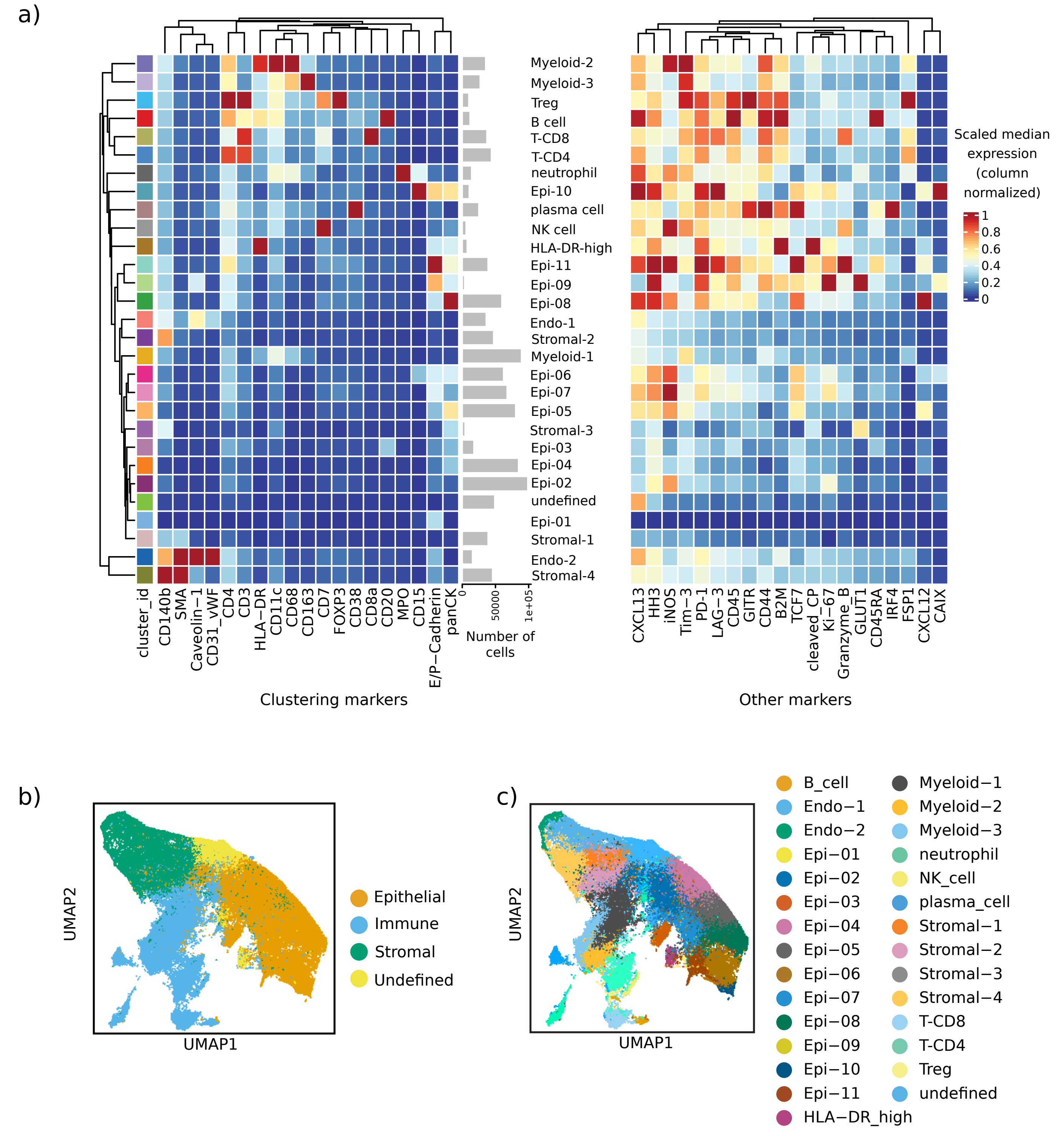

Supplementary figure 6. Initial clustering of single-cell data from T cell panel to identify epithelial, immune, and stromal cells. a) Heatmap of cluster-level mean expression. Graph-based clustering with Louvain community detection (k=5, jaccard) was used over all the cells (n = 87 patients, n_cells = 1,068,958, 695 images) calculated over 99th-percentile range normalized values using key markers to distinguish main cell types (CD140b, SMA, Caveolin-1, CD31_vWF, CD4, CD3, HLA-DR, CD11c, CD68, CD163, CD7, FOXP3, CD38, CD8a, CD20, MPO, CD15, E/P-Cadherin, panCK). Middle: Bar chart with the number of cells in each cluster. Right: Heatmap of cluster-level mean expression for markers not used for clustering. b) Single-cell level UMAP calculated over the same markers as used for clustering using the 99th percentile range normalized intensity values. From images with <300 cells all cells were used, otherwise 300 cells were randomly sampled. Cells are colored by cell class annotation (n = 87 patients, n_cells= 199 822, 681 images). c) Same as (b) with cells colored by cluster ID.

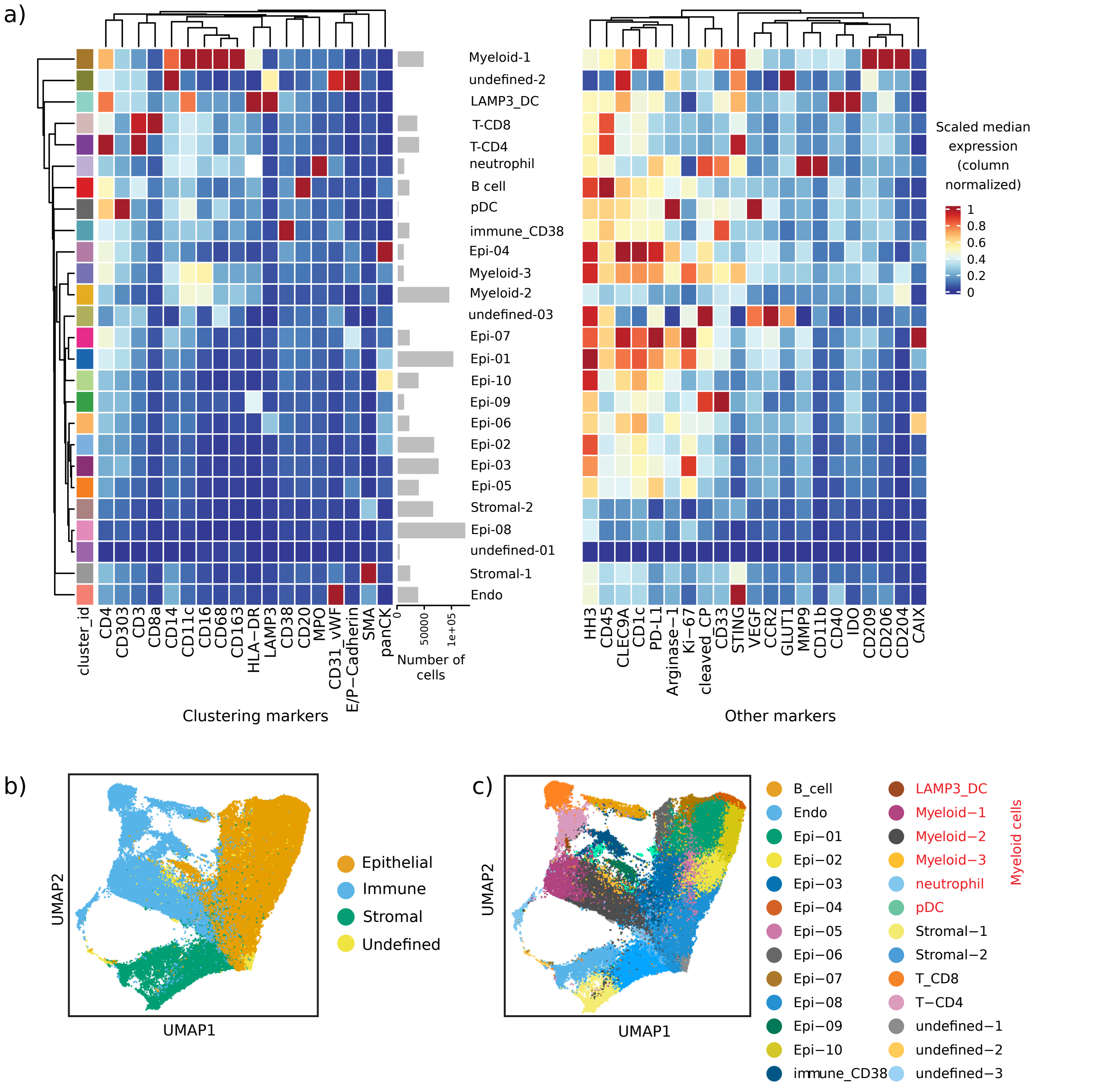

Supplementary figure 7. Initial clustering of single-cell data from myeloid cell panel to identify epithelial, immune, and stromal cells. a) Heatmap of cluster-level mean expression. Graph-based clustering with Louvain community detection (k=5, jaccard) was used over all the cells (n = 87 patients, n_cells = 989,897, 681 images) calculated over 99th-percentile range normalized values using key markers to distinguish main cell types (CD4, CD303, CD3, CD8a, CD14, CD11c, CD16, CD68, CD163, HLA-DR, LAMP3, CD38, CD20, MPO, CD31_vWF, E/P-Cadherin, SMA, panCK). Middle: Bar chart with number of cells in each cluster. Right: Heatmap of cluster-level mean expression for markers not used for clustering. b) Single-cell level UMAP calculated over the same markers as used for clustering using the 99th percentile range normalized intensity values. From images with <300 cells all cells were used, otherwise 300 cells were randomly sampled. Cells are colored by cell class annotation (n = 87 patients, n_cells= 196 734, 681 images). c) Same as (b) with cells colored by cluster ID.

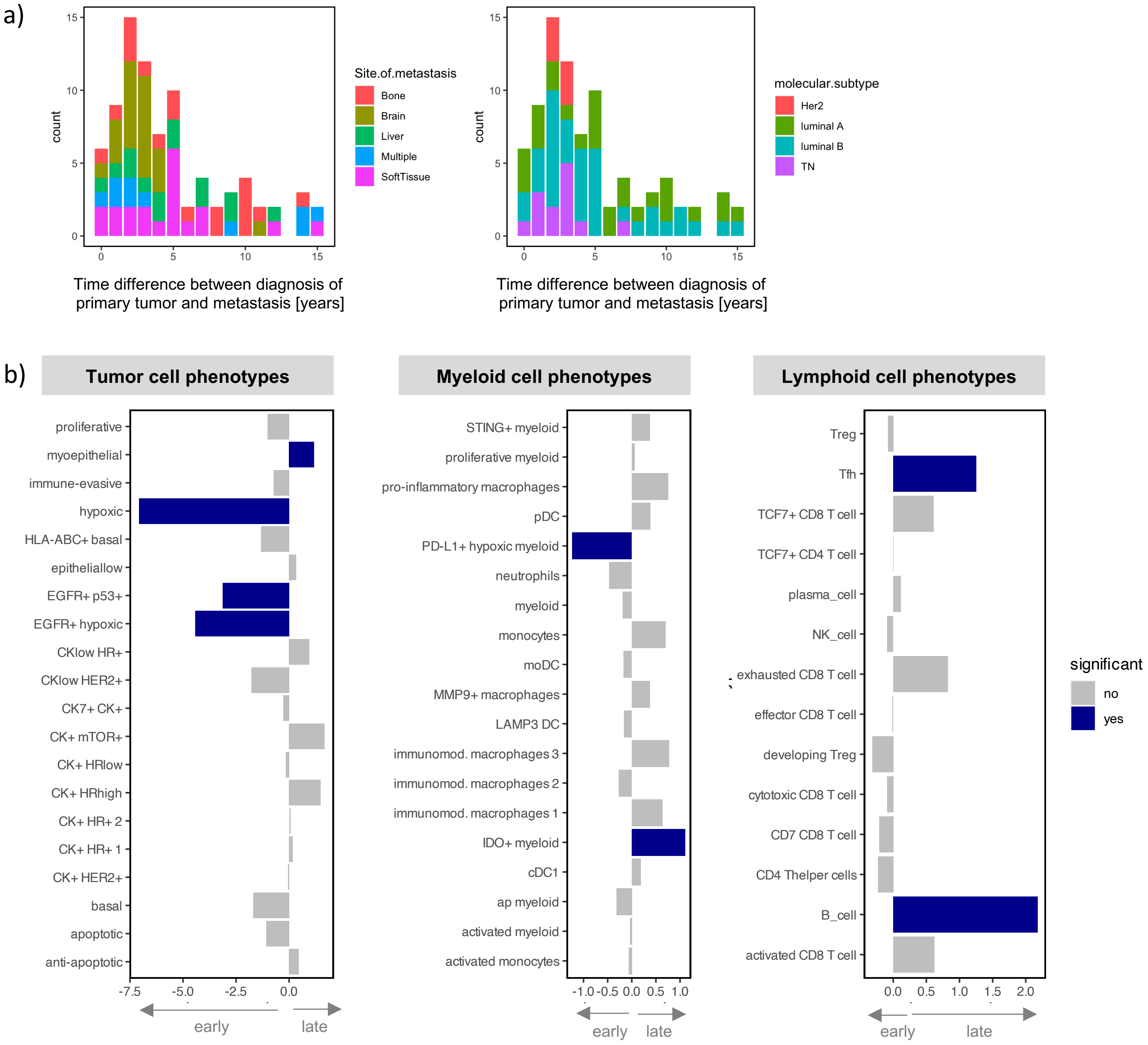

Supplementary figure 8. Comparison of primary tumor composition in patients with early and late relapse. a) Barplot illustrating the distribution of patients based on the duration (in years) between diagnosis of the primary tumor and occurrence of metastasis. The color-coded bars represent different metastatic sites (left) and molecular subtypes (right). b) Cell phenotypic cluster enrichment in primary tumors with late relapses compared to primary tumors with early relapses by differential abundance testing. Blue bars display significantly enriched or decreased (FDR < 0.05; multiple testing correction with Benjamin-Hochberg method, molecular subtype as co-variate) results with bar length indicating the log-transformed fold change. Early and late relapse were hereby classified based on a median split of the patients.

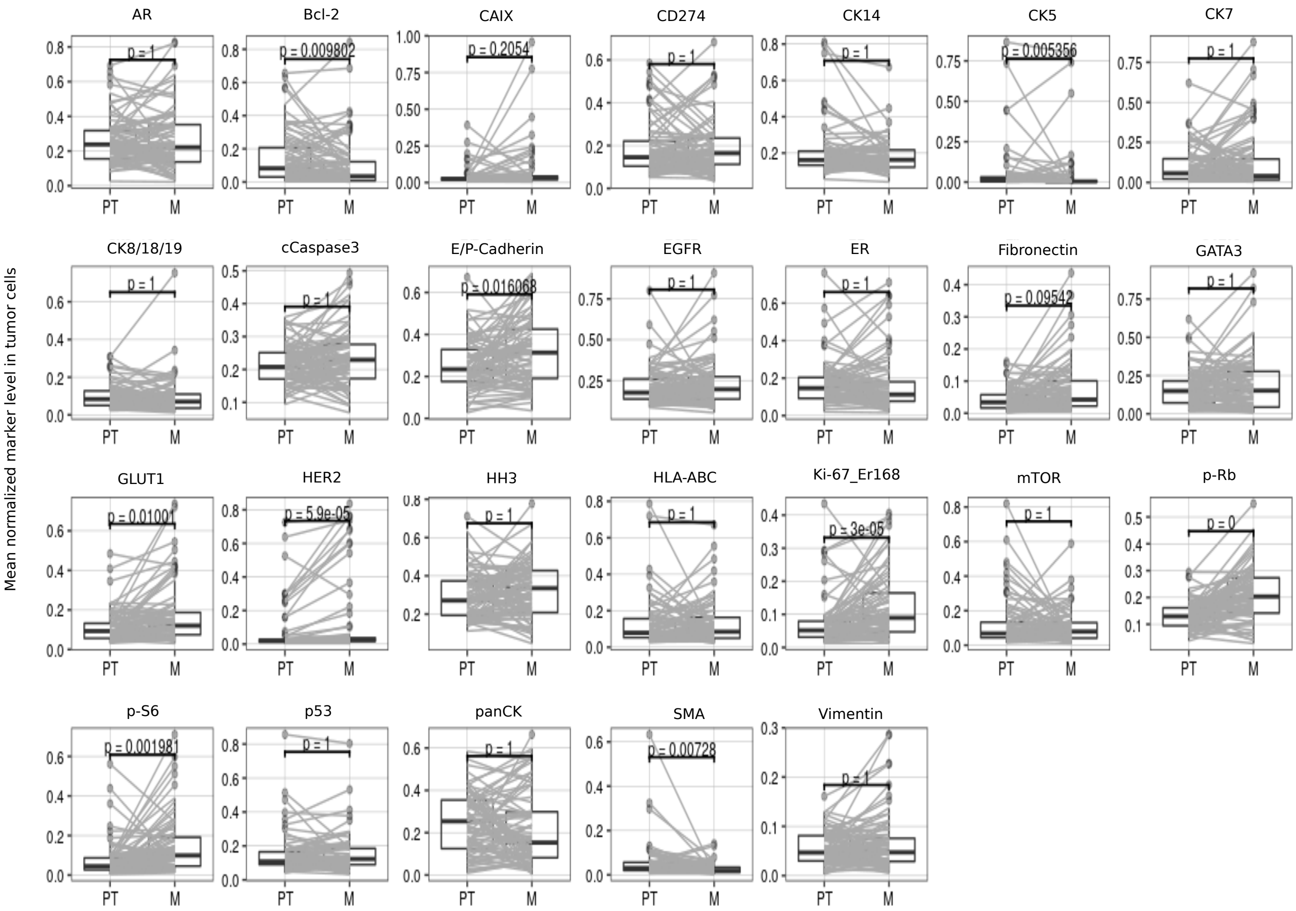

Supplementary figure 9. Mean marker expression in primary tumors and metastatic sites. Mean expression values across all tumor cells for epithelial markers in primary tumor and metastatic samples are shown pairwise for all patients (n=79 patients). P values are shown for each comparison (paired Wilcoxon test with Bonferroni correction). For all panels, all marker levels were normalized to the 99 percentile.

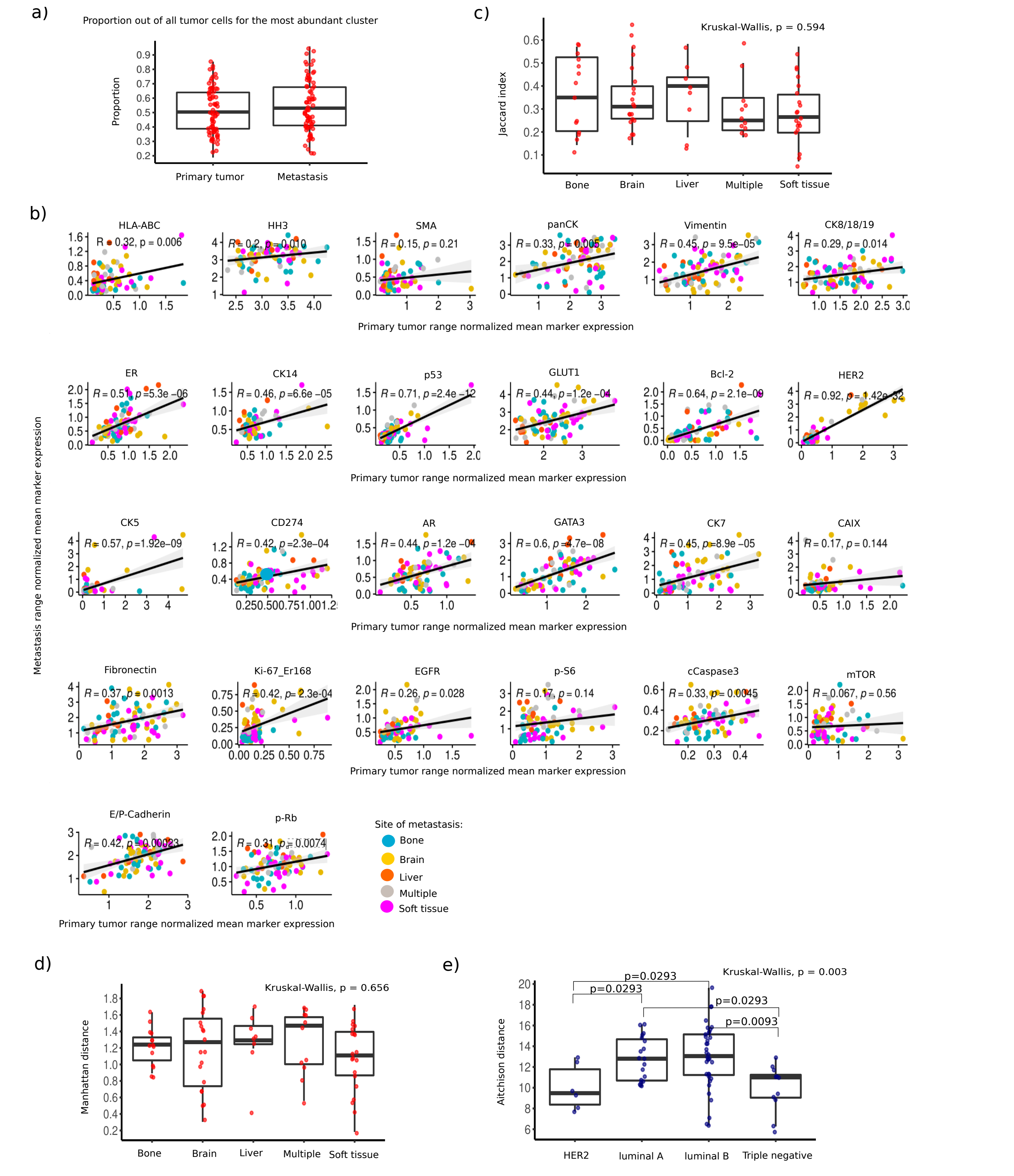

Supplementary figure 10. Similarity analysis of tumor cell phenotypes and marker expression between primary tumors and metastases. a) Box plot of the proportion of the most abundant phenotypic cluster in primary tumor and metastatic samples across patients (n=73 patients). b) Pearson correlation of mean marker expression between the primary tumor and metastatic samples for each patient (n=73 patients). Samples from patients with multiple metastatic sites were aggregated across the sites. Adjusted p-values with the Benjamin Hochberg method are displayed. The gray shadow shows the confidence interval. Marker expression values have been arcsinh transformed. c) Pairwise Jaccard index for matched samples grouped by the site of metastasis. Jaccard index was calculated for each primary tumor and metastatic sample per patient over binarized cluster abundances. A cluster was considered present if it had abundance >=1%, otherwise absent. d) Pairwise Manhattan distance for matched samples grouped by the site of metastasis. Manhattan distance was calculated over the abundances for each primary tumor and metastatic sample per patient. e) Pairwise Aitchison distance for matched samples grouped by molecular subtype followed by grouped pairwise Wilcoxon test with Benjamin Hochberg correction.

a)

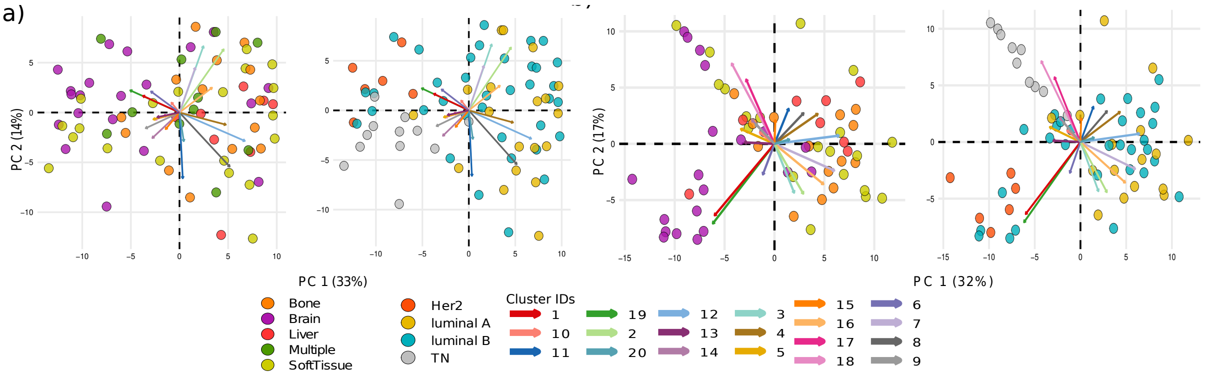

PERMANOVA on proportional abundance for primary tumors only:

	DF	SumsOfSqs	MeanSqs	F	R2	p(adj)
Molecular subtype (1)	3	2776.2	925.4	7.5027	0.24134	0.001
Site of metastasis (2)	4	727.5	181.89	1.4747	0.06325	0.064
Interaction(1x2)	7	845.6	120.79	0.9793	0.07351	0.469
Residuals	58	7153.8	123.34	0.6219		

PERMANOVA on proportional abundance for metastasis only:

	DF	SumsOfSqs	MeanSqs	F	R2	p(adj)
Molecular subtype (1)	3	2511.3	837.11	8.4505	0.27787	0.001
Site of metastasis (2)	3	777.6	259.2	2.6165	0.08604	0.001
Interaction(1x2)	5	795.9	159.18	1.6069	0.08806	0.018
Residuals	50	4953	99.06		0.54803	

c)

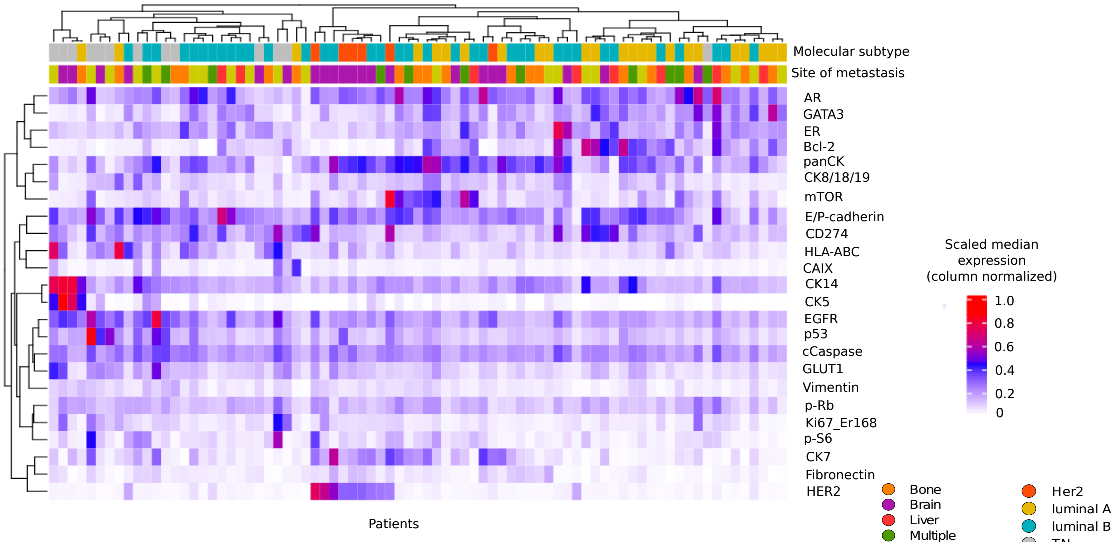

d)

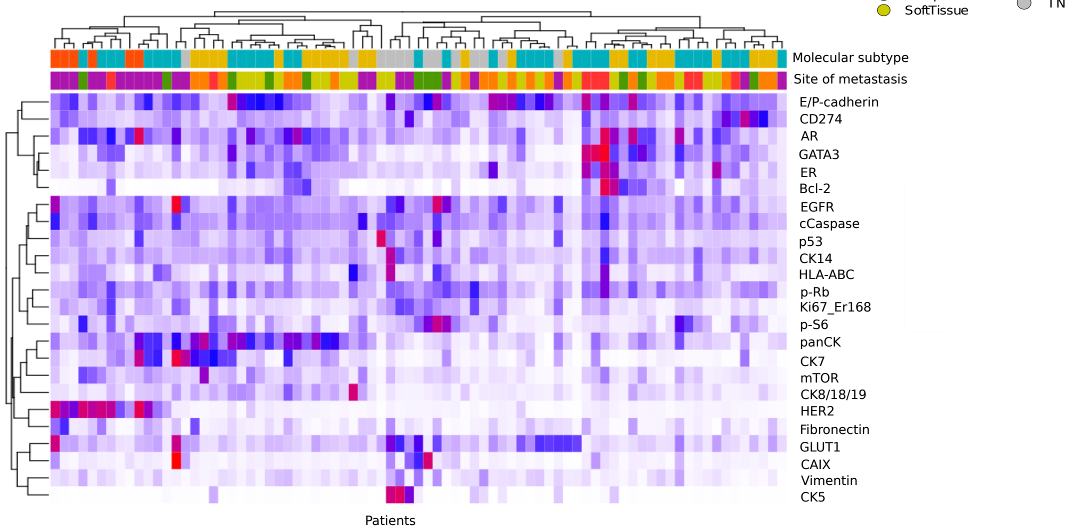

Supplementary figure 11. Tumor phenotypic cluster composition by metastatic site and molecular subtype and hierarchical clustering of patients based on mean marker expression level in tumor cells a) Comparison of phenotypic cluster abundances in primary tumor samples that metastasize to different sites, or of different molecular subtypes. The table shows the results of a PERMANOVA test which determines if the centers of groups are significantly different. The PCA was performed over phenotypic cluster abundances (centered log-ratio transformed abundances) per patient sample (n=73 patients); each dot represents a single primary tumor patient sample, the molecular subtype, and site of metastasis are indicated. b) As in (a) for metastatic samples(n=62 patients). c) Heatmap of mean marker expression per patient in epithelial cells from the primary tumor samples (n=79 patients). Patient IDs are on the x axis, and markers are on the y axis. d) Same as in (c) for metastatic samples (n=79 patients). Samples from patients with multiple metastatic sites were aggregated across the samples. Pearson correlation as distance measure and complete linkage was used for hierarchical clustering for both heatmaps.

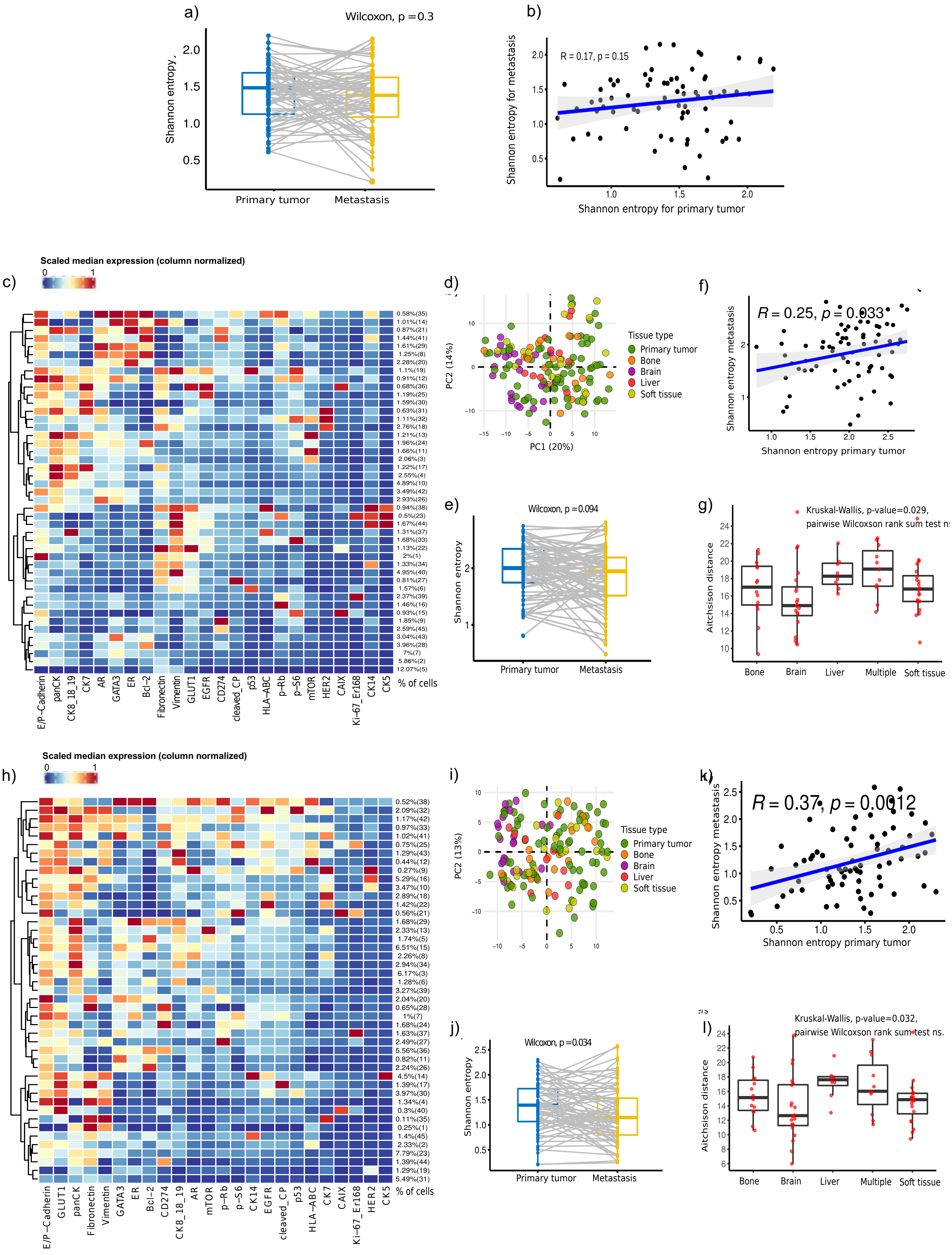

a)
Wilcoxon, p = 0.3
Shannon entropy
Primary tumor
Metastasis
b)
R = 0.17, p = 0.15
Shannon entropy for metastasis
Shannon entropy for primary tumor
c)
Scaled median expression (column normalized)
E/P−Cadherin
panCK
CK8_18_19
CK7
AR
GATA3
ER
Bcl−2
Fibronectin
Vimentin
GLUT1
EGFR
CD274
cleaved_CP
p53
HLA−ABC
p−Rb
p−S6
mTOR
HER2
CAIX
Ki−67_Er168
CK14
CK5
% of cells
d)
PC2 (14%)
PC1 (20%)
Tissue type
Primary tumor
Bone
Brain
Liver
Soft tissue
e)
Wilcoxon, p = 0.094
Shannon entropy
Primary tumor
Metastasis
f)
R = 0.25, p = 0.033
Shannon entropy metastasis
Shannon entropy primary tumor
g)
Kruskal-Wallis, p-value=0.029,
pairwise Wilcoxson rank sum test ns
Aitchsison distance
Bone
Brain
Liver
Multiple
Soft tissue
h)
Scaled median expression (column normalized)
E/P−Cadherin
GLUT1
panCK
Fibronectin
Vimentin
GATA3
ER
Bcl−2
CD274
CK8_18_19
AR
mTOR
p−Rb
p−S6
CK14
EGFR
cleaved_CP
p53
HLA−ABC
CK7
CAIX
Ki−67_Er168
HER2
CK5
% of cells
i)
PC2 (13%)
Tissue type
Primary tumor
Bone
Brain
Liver
Soft tissue
j)
Wilcoxon, p = 0.034
Shannon entropy
Primary tumor
Metastasis
k)
R = 0.37, p = 0.0012
Shannon entropy metastasis
Shannon entropy primary tumor
l)
Kruskal-Wallis, p-value=0.032,
pairwise Wilcoxson rank sum test ns.
Aitchsison distance
Bone
Brain
Liver
Multiple
Soft tissue

Supplementary figure 12. Phenotypic analysis of tumor cells and their heterogeneity with alternative clustering approaches. a) Phenotypic heterogeneity (Shannon index) based on the cluster abundances for each patient in primary tumors vs metastatic samples. Shannon index was calculated over the phenotypic cluster abundances for each patient sample. The Wilcoxon signed-rank test was used for determining significant differences between groups. b) Pearson correlation between phenotypic heterogeneity (Shannon index) for primary tumor and metastatic samples. c) Left: Heatmap of median expression of epithelial cell clusters with cells pooled across all samples (n=87, n_cells = 559,953). K-means clustering with range normalized values with k=45 was used for single-cell clustering. Euclidean distance with Ward-D2 linkage was used for the hierarchical clustering of rows. Right: Bar plot with cell counts in each cluster. d) PCA over centered log-ratio transformed phenotypic cluster abundances (with pseudo count 1) per patient sample (n=73). e) Phenotypic heterogeneity (Shannon entropy) based on the cluster abundances for each patient in primary tumors vs metastatic samples. Shannon entropy was calculated over the phenotypic cluster abundances for each patient sample. The Wilcoxon signed-rank test was used for determining significant differences between groups. f) Pearson correlation between phenotypic heterogeneity (Shannon entropy) for primary tumor and metastatic samples. g) Pairwise Aitchison distance for matched samples grouped by site of metastasis. Aitchison distance was calculated as Euclidean distance between the centered log-ratio transformed phenotypic abundances for each primary tumor and metastatic sample per patient. h) Left: Heatmap of median expression of epithelial cell clusters with cells pooled across all samples (n=87, n_cells = 559,953). Graph-based clustering with Jaccard index and Louvain community detection over range normalized values with k=20 was used for single-cell clustering. Euclidean distance with Ward-D2 linkage was used for the hierarchical clustering of rows. Right: Bar plot with cell counts in each cluster. i) Same as (d) but using graph-based clusters. j) Same as e) for graph-based clusters. k) Same as f) for graph-based clusters. l) Same as g) for graph-based clusters.

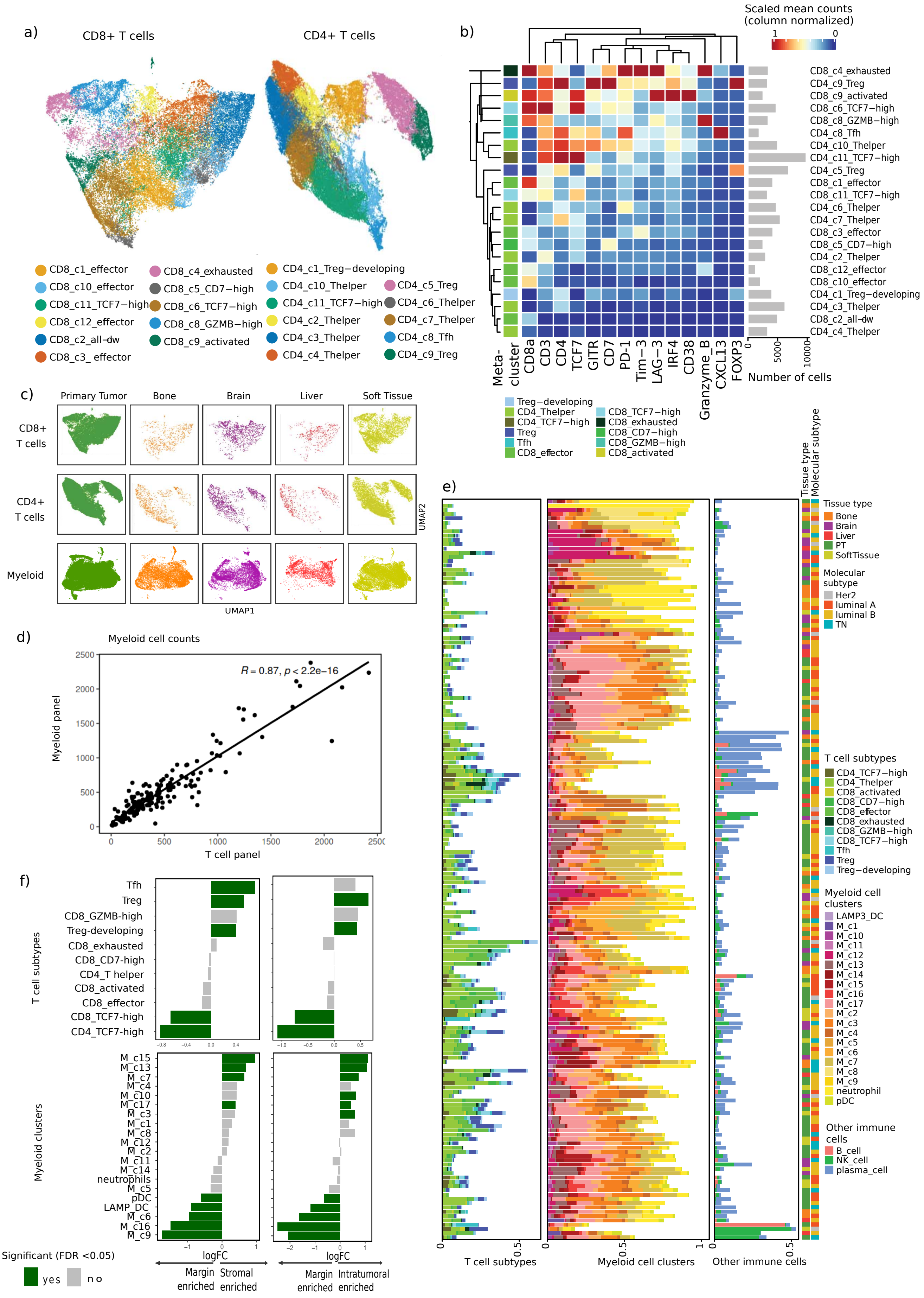
a)
CD8+ T cells
CD4+ T cells
CD8_c1_effector
CD8_c10_effector
CD8_c11_TCF7−high
CD8_c12_effector
CD8_c2_all−dw
CD8_c3_ effector
CD8_c4_exhausted
CD8_c5_CD7−high
CD8_c6_TCF7−high
CD8_c8_GZMB−high
CD8_c9_activated
CD4_c1_Treg−developing
CD4_c10_Thelper
CD4_c11_TCF7−high
CD4_c2_Thelper
CD4_c3_Thelper
CD4_c4_Thelper
CD4_c5_Treg
CD4_c6_Thelper
CD4_c7_Thelper
CD4_c8_Tfh
CD4_c9_Treg
b)
Scaled mean counts (column normalized)
Meta-cluster
CD8a
CD3
CD4
TCF7
GITR
CD7
PD-1
Tim−3
LAG−3
IRF4
CD38
Granzyme_B
CXCL13
FOXP3
Number of cells
Treg−developing
CD4_Thelper
CD4_TCF7−high
Treg
Tfh
CD8_effector
CD8_TCF7−high
CD8_exhausted
CD8_CD7−high
CD8_GZMB−high
CD8_activated
c)
Primary Tumor
Bone
Brain
Liver
Soft Tissue
CD8+ T cells
CD4+ T cells
Myeloid
UMAP1
UMAP2
d)
Myeloid cell counts
R = 0.87, p < 2.2e−16
Myeloid panel
T cell panel
e)
T cell subtypes
Myeloid cell clusters
Other immune cells
Tissue type
Molecular subtype
Bone
Brain
Liver
PT
SoftTissue
Her2
luminal A
luminal B
TN
LAMP3_DC
neutrophil
pDC
B_cell
NK_cell
plasma_cell
f)
Tfh
Treg
CD8_GZMB-high
Treg-developing
CD8_exhausted
CD8_CD7-high
CD4_T helper
CD8_activated
CD8_effector
CD8_TCF7-high
CD4_TCF7-high
Myeloid clusters
neutrophils
pDC
LAMP_DC
logFC
Margin enriched
Stromal enriched
Intratumoral enriched
Significant (FDR <0.05)
yes
no

Supplementary figure 13. A high-resolution compositional map of matched primary and metastatic TIMEs. a) UMAP visualization of CD8+ (left) and CD4+ T cells (right) from the T cell panel dataset, colored by T cell phenotypic clusters. b) Heatmap showing the column-normalized mean expression of T cell-relevant markers across T cell subclusters. c) UMAP visualization of CD8+ T cells, CD4+ T cells, and myeloid cells as in Fig. 4a and 4c, split by tissue type. d) Pearson correlation between per-patient myeloid cell counts from images stained with the immune or the myeloid panels. e) Stacked bar plots showing the full TIME composition of each sample (y axis), split up into T cell subtypes, myeloid cell subclusters, and other immune cells. Only intratumoral images were included for this analysis, and images of the same sample were pooled. The molecular subtype and metastatic site (tissue type) of each sample are indicated. logFC = log2 Fold-Change. f) Top: Barplots showing the log2-fold abundance changes of different T cell subtypes (relative to total T cells) in stromal (left) and intratumoral regions (right) compared to margin regions of primary tumors. The design of the differential abundance model accounts for patient ID. Bottom: Barplots showing the log2-fold abundance changes of different myeloid clusters (relative to total myeloid cells) in stromal (left) and intratumoral regions (right) compared to margin regions of primary tumors. The design of the differential abundance model accounts for patient ID.

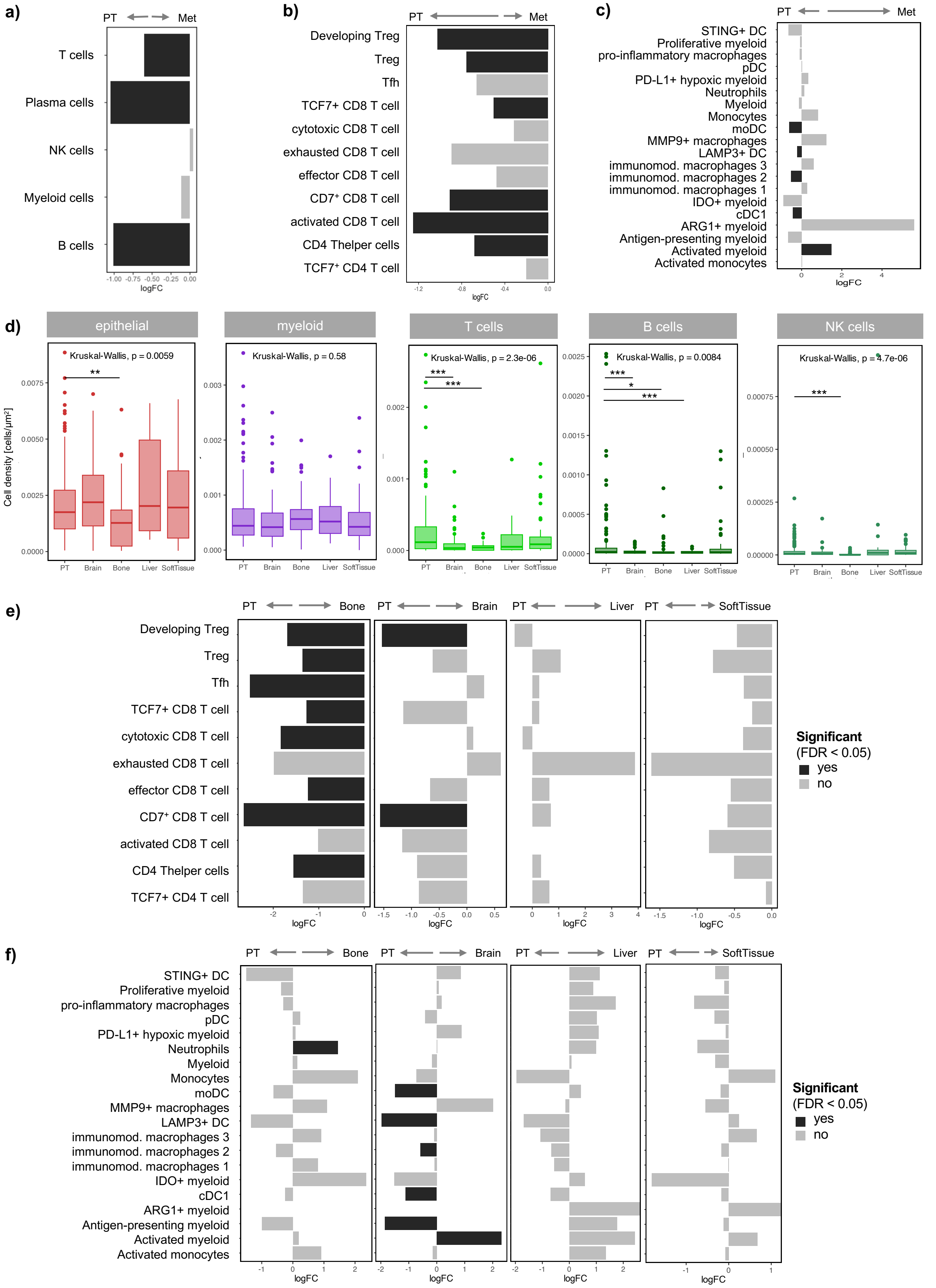

Supplementary Figure 14. Immune cell subtype densities in primary tumors and metastatic samples. a-c) Changes in immune cell type densities in primary tumors (PT) or metastasis (Met) determined via Mann-Whitney U testing using paired design. Bar plot shows the log2-fold abundance changes (n=76 patients). d) Box plots display the density of each cell type in the primary tumor and the indicated metastatic location. Data are shown per intratumoral image. e-f) Changes in T cell (e) and myeloid (f) subtype densities in primary tumors (PT) or metastasis (Met) determined via Mann-Whitney U testing using paired design. Bar plot shows the log2-fold abundance changes (n=76 patients).

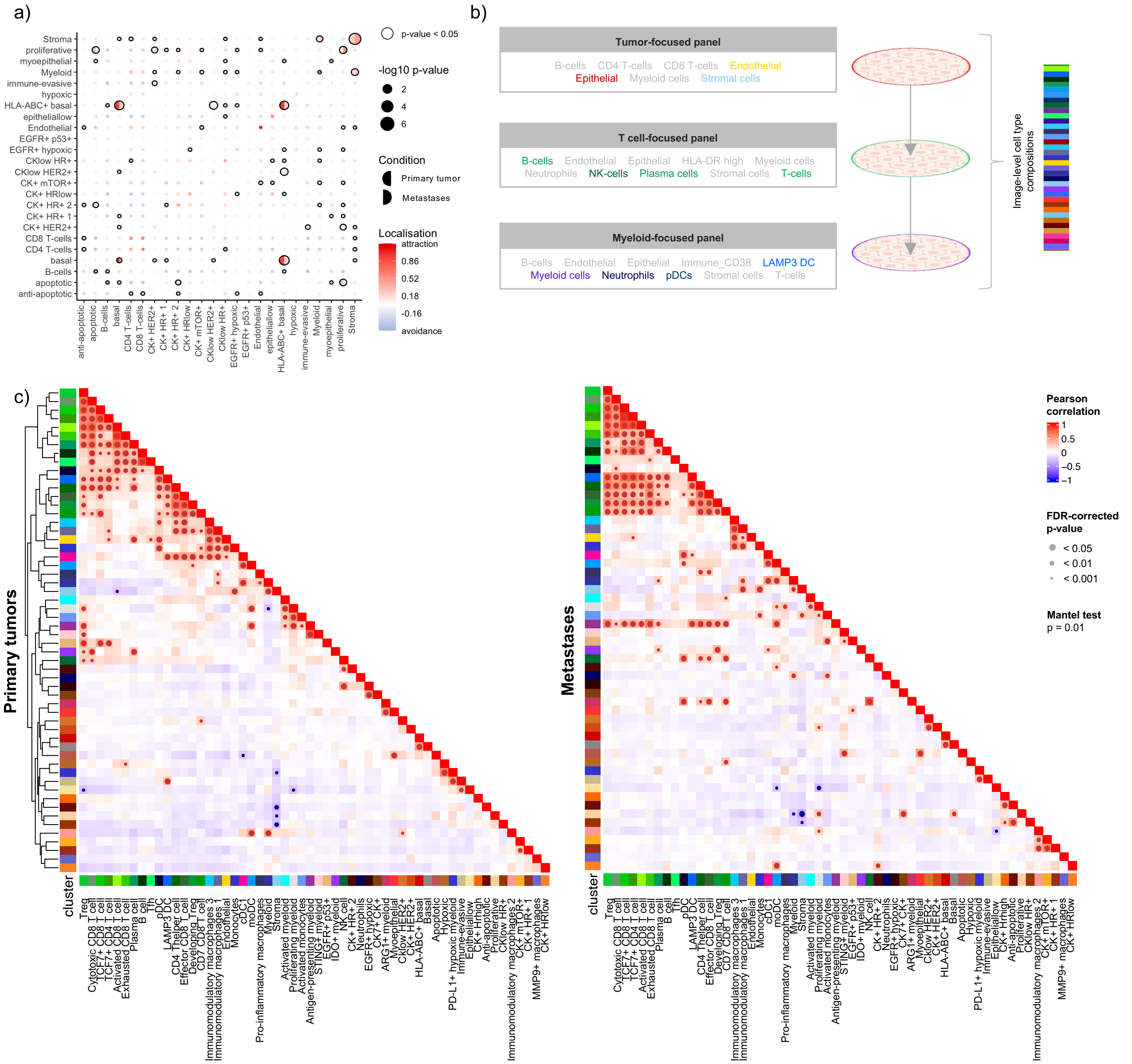

Supplementary figure 15. Cell type correlations in primary tumors and metastases. a) Bubble plot depicts interaction/avoidance changes between primary tumors (left) and metastases (right) with a black outline indicating significance. This analysis was done using only the tumor panel. Circle size correlates with the -log10 p-value. b) Schematic representation depicting the process of extracting distinct cell types from three antibody panels, followed by their integration into a relative composition at the image level. c) Heatmaps illustrating the Pearson correlation coefficients between relative cell type frequencies across intratumoral regions in primary tumors (left) and distant metastases (right) on an image level. FDR-corrected significant correlations are denoted by a circle.

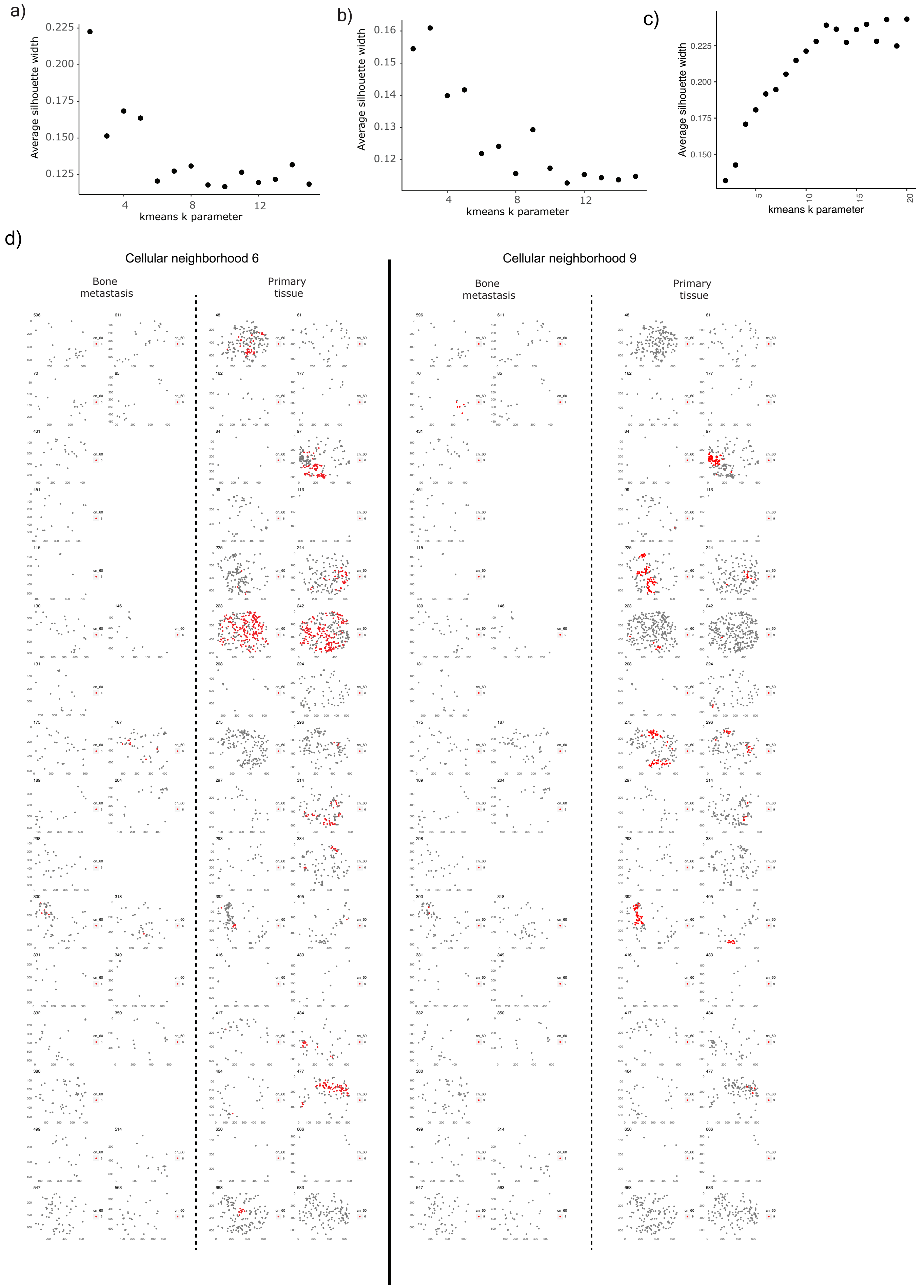

a)
Average silhouette width
kmeans k parameter
b)
Average silhouette width
kmeans k parameter
c)
Average silhouette width
kmeans k parameter
d)
Cellular neighborhood 6
Bone metastasis
Primary tissue
Cellular neighborhood 9
Bone metastasis
Primary tissue

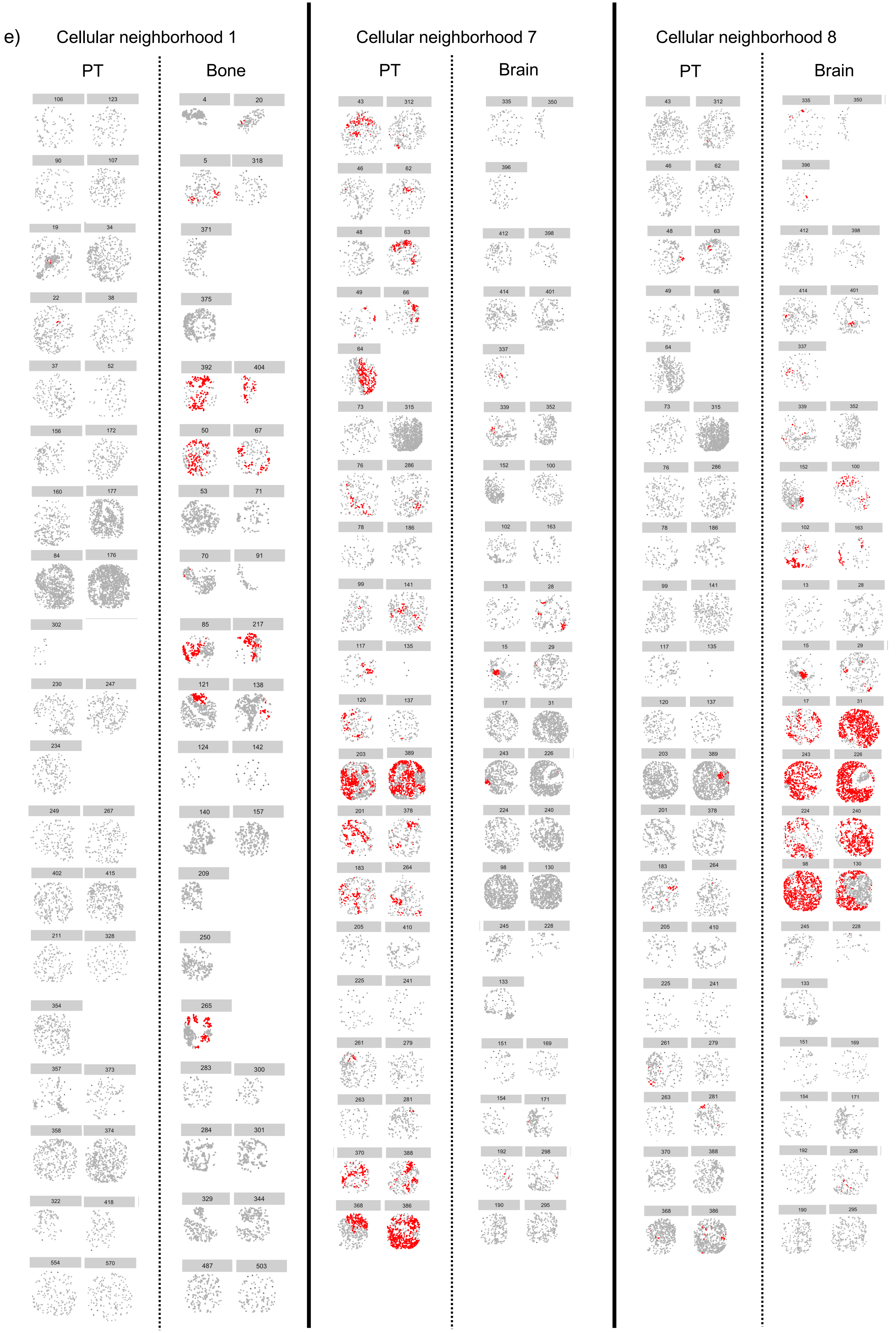

e)
Cellular neighborhood 1
PT
Bone
Cellular neighborhood 7
PT
Brain
Cellular neighborhood 8
PT
Brain

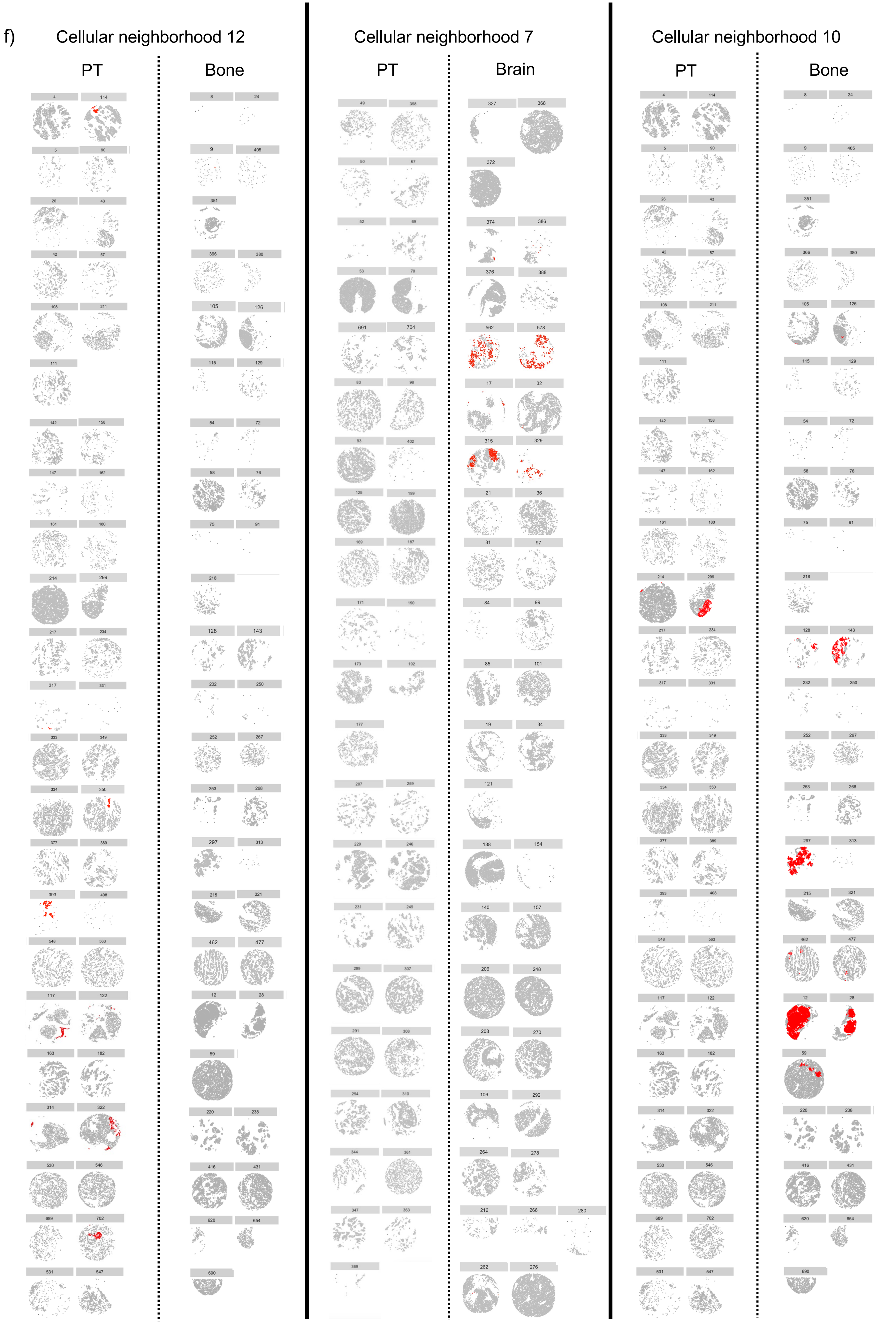
f)
Cellular neighborhood 12
PT
Bone
Cellular neighborhood 7
PT
Brain
Cellular neighborhood 10
PT
Bone

Supplementary figure 16. Technical validation and visual assessment of the cellular neighborhood analysis approach. a) The centered log-ratio transformed counts of T cell frequencies in the 60µm neighborhood around each T cells were clustered with kmeans algorithm 14 times with increasing k (2 to 15). For each clustering result the average silhouette width is calculated and visualized. For k equal to 14 the silhouette width shows a local maximum indicating the optimal k. b) The same procedure as in a) was applied to the identify the optimal k when clustering myeloid cell frequencies within the 60µm neighborhood of each myeloid cell. The parameter sweep shows the highest silhouette for k equals to 9. c) The same procedure as in a) was applied to identify the optimal k when clustering epithelial cell frequencies within the 60 µm neighborhood of each tumor cell. The parameter sweep shows the highest silhouette for k equals to 12. d) Visual assessment of differentially abundant T cell neighborhoods (6 and 9) between primary tissue and bone metastasis. Each individual panel represents a single image and images in one row belong to one patient. Each dot represents the centroid of an individual T cell. Red cells belong to the cellular neighborhood of interest. e) Visual assessment of differentially abundant myeloid cell neighborhoods for the indicated tissue comparisons. As in d) each panel represents one image and images in one row belong to one patient. Each dot represents the centroid of an individual myeloid cell in their spatial location. Red cells belong to the cellular neighborhood of interest. f) Exemplary visual assessment of differentially abundant epithelial cell neighborhoods of interest. As in d) each panel represents one image and images in one row belong to one patient. Each dot represents the centroid of an individual myeloid cell in their spatial location. Red cells belong to the cellular neighborhood of interest.
